# Dynamic Response Spectroscopy: An Emergentist Framework for Multi-Timescale Catalytic Interfacial Dynamics

**DOI:** 10.1021/acscatal.5c05171

**Published:** 2025-11-07

**Authors:** Daniel Sinausia, Florian Meirer, Anatoly I. Frenkel, Charlotte Vogt

**Affiliations:** † Schulich Faculty of Chemistry and Resnick Sustainability Center for Catalysis, 26747 Technion—Israel Institute of Technology, Haifa 3200002, Israel; ‡ Inorganic Chemistry and Catalysis, Institute for Sustainable and Circular Chemistry, Utrecht University, Utrecht 3584 CG, The Netherlands; § Department of Materials Science and Chemical Engineering, 12301 Stony Brook University, Stony Brook, New York 11794, United States; ∥ Division of Chemistry, Brookhaven National Laboratory, Upton, New York 11973, United States

**Keywords:** dynamic response spectroscopy, DRS, interfacial dynamics, time-resolved spectroscopy, dimensionality reduction, nonlinear system dynamics

## Abstract

The interfaces that govern catalytic reactivity exhibit complex, often coupled, multi-timescale behavior arising from the dynamic organization of ions, solvent molecules, and adsorbates. This complexity is especially pronounced in electrochemical systems where classical models describing the *Stern* or *diffuse double* layers are, in practice, neither static nor ideally defined backgrounds, but active, dynamic contributors to catalytic function. Nevertheless, most electrochemical and spectroscopic probes rely on assumptions of linearity or time invariance, (implicitly) limiting their ability to resolve such intricacies. In this Perspective, we formalize and expand on Dynamic Response Spectroscopy (DRS), a framework that leverages temporally structured perturbations and time-resolved spectroscopic detection to disentangle overlapping, and potentially coupled, nonlinear interfacial dynamics, including non-Faradaic processes and other dynamics not directly reflected in product turnover. While we focus on electrochemical systems as our primary example, the DRS framework is in principle applicable to all (catalytic) systems exhibiting complex interfacial dynamics. We introduce a generalized simulation approach to model spectrotemporal responses to modulation, enabling systematic evaluation of component (elementary reaction and process) retrievability across varying coupling topologies and kinetic regimes. We illustrate the capabilities of DRS using both synthetic systems and, as a case study, experimental operando ATR-SEIRAS measurements during CO_2_ electroreduction on copper. The results demonstrate how DRS can uncover solvent dynamics, charging delays, and memory effects that elude current-only, single frequency, or modality analyses. Rather than imposing predefined mechanistic assumptions, DRS allows the system’s natural dynamical structure to emerge. We discuss the conceptual implications and practical considerations for implementing DRS across catalytic systems. By acknowledging time-domain complexity, DRS offers an alternative axis of mechanistic insight into the emergent behaviors that govern catalytic activity, selectivity, and stability.

## Graphical abstract

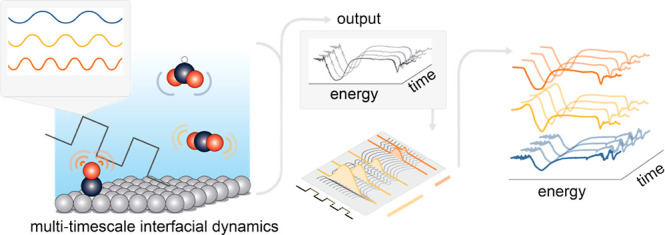

## Introduction

Catalytic processes at solid–liquid and solid-gas interfaces are key enablers in the transition to a more sustainable, circular society. At their core lies the reactive interface, whether in thermocatalysis, photocatalysis, or electrocatalysis; a nanoscopic, time-evolving region where potential, composition, and structure continuously reshape one another. For example, the electrified interface relevant to electrochemical systems hosts a hierarchy of interconnected phenomena, from fast double-layer charging and adsorbate dynamics to slower ion migration, diffusion, and solvation dynamics.^1–4^

These processes span orders of magnitude in time, space, and energy, and they are rarely independent. Indeed, nonlinear coupling, memory effects, and, for example, sensitivity to local gradients (e.g., pH, hydration structure, interfacial fields) are intrinsic features of all electrochemical systems,^5,6^ such that electrochemically probing one component of this intertwined network of multiphysical processes may perturb others.^7^ For these reasons, resolving the full complexity of electrochemical interfaces requires more than high spatial or spectral resolution. It requires an explicit framework in which one can interrogate and resolve overlapping dynamically coupled component behavior with physicochemical resolution. This need is especially explicit in realistic systems, where polycrystallinity, electrolyte complexity, and porous structures create interfacial microenvironments that can be particularly heterogeneous and dynamically responsive, eluding current theoretical models, which is a critical frontier in modern electrocatalysis.^8,9^ Although here we focus on electrochemical systems as a representative case, similar challenges arise in heterogeneous and photocatalytic interfaces, where solvent dynamics, adsorbate restructuring, and surface transformations can likewise govern activity and selectivity.

(Spectro)­electrochemical methods have greatly advanced our understanding of interfacial structure and reactivity, but major challenges remain, especially in resolving emergent coupled dynamics. Many available tools collapse time-dependent behavior through averaging or are limited to scalar observables, such as current or potential. These observables represent aggregate signals that conflate multiple underlying processes, often rendering individual contributions inseparable. As a result, reductionist approaches^10^—such as electrochemical impedance spectroscopy (EIS)—are typically employed, wherein each component is probed sequentially using single-frequency perturbations under the assumption of stationarity (time invariance, i.e., where system variables are constant over time). This inherently excludes the possibility of capturing emergent or time-evolving interactions among the system components. But even advanced electrochemical perturbation strategies like Fourier-transformed AC voltammetry or nonlinear EIS (NL-EIS)^11–13^ offer limited mechanistic speciation compared to photon-based techniques. Unfortunately, poor signals in the latter often necessitate long averaging periods,^14,15^ which offers molecular insight at the cost of dynamic context. The consequences of these limitations are not only methodological but catalytic, as critical couplings between solvents, adsorbates, and charge carriers can dictate turnover frequencies and product distributions in electrocatalysis, yet remain hidden from conventional probes.

Some frameworks have attempted to bridge this gap. Particularly in heterogeneous thermocatalysis, modulated excitation spectroscopy (MES) combined with phase-sensitive detection (PSD) has demonstrated success in demodulating weak spectroscopic signals from complex systems.^3,16–20^ However, such approaches are typically phase-locked and optimized for stationary, fully reversible, periodic responses as they assume steady-state or equilibrated systems.^21^ Such approaches fail when system dynamics deviate from the modulation period, as in the case of aperiodic, nonlinear, or irreversibly evolving processes, which play an even larger role in heterogeneous electrocatalysis which are intrinsically driven, out of equilibrium systems.^22^ These limitations constrain our ability to interrogate the complex dynamic interplay of interfacial modes, such as those present at the electrode–electrolyte interface, which are crucial to fully understand, and thus design, activity, selectivity, and stability.

We argue that we must (re)­consider how we design perturbations (modulations), what observables we prioritize, which assumptions we are willing to relax, and what we must be wary of when aiming to characterize these complex, coupled interfacial dynamics. We build upon the powerful existing methods that have yielded much of our current understanding, to enable emergentist approaches that can observe real-world dynamic complexity, and to do that we must highlight where excessive simplification creates *blind spots*. We recently introduced, and now formalize, dynamic response spectroscopy (DRS).^21^ DRS was developed with catalysis in mind; by disentangling multi-timescale dynamics at interfaces, it allows us to connect transient physicochemical changes directly to catalytic activity and selectivity. It thus complements existing tools and provides a framework that is equally relevant to electrocatalysis as to other (catalytic) systems where emergent dynamics shape performance. DRS is a general framework designed to interrogate time-dependent interfacial phenomena through temporally structured perturbations, broadband time-resolved spectroscopy, and model-lean, or even model-free, analysis. That is, rather than presupposing linear, reversible, or synchronous system behavior, we treat the interface as a multiple-input, multiple-output (MIMO) system,^23,24^ aiming to enable a category of experiments that track how entire classes of interconnected spectral features evolve as a function of both time and applied waveform. This allows investigation of otherwise inaccessible couplings, emergent modes, and other nontrivial dynamic behavior. By treating the interface as a dynamical system rather than a static boundary, DRS provides a route to uncover mechanistic principles that are broadly applicable across catalysis, from electric double layer dynamics in CO_2_ electroreduction to thermocatalytic surface restructuring.

In this work, we introduce a modeling framework where spectrotemporal matrices are simulated with varying degrees and types of coupled components responding to perturbations to systematically analyze the resolvability of complex coupled nonlinear spectral-dynamic systems. Thereby, we specifically address a long-standing challenge in operando catalysis; the deconvolution of simultaneously active, time-variant, coupled interfacial processes that yield spectroscopically overlapping responses. We discuss a few key cases by analyzing the retrievability of system response functions under varying degrees of complexity via (non)­linearity and coupling by comparing various data mining approaches. We also explore how waveform characteristics such as frequency content and amplitude affect excitability and process separability. We emphasize the power of cross-modal broadband observables (such as certain vibrational or electronic spectroscopies) in disentangling time-resolved responses. The right cross-modal observable to a structured waveform electrochemical perturbation allows one to access state variables invisible to scalar electrochemical detection alone, such as current. We discuss and provide practical separability criteria for spectral and temporal disentanglement.

Building on this, we show how dimensionality reduction techniques (e.g., principal component analysis, PCA) can isolate spectral-temporal modes and how newer approaches, including nonlinear manifold learning, autoencoders (AE), and dynamic mode decomposition (DMD), can extend interpretability in regimes where linear approaches can fail. As illustrated in Figure 1A, DRS bridges the conceptual gap between classic electrochemical probes, spectroscopy, and emergent systems and information theory. When systematically designed and rigorously employed, the approach allows one to transform the interface from a black box to a dynamic system whose internal modes can be systematically perturbed, observed, and ultimately understood. DRS does not aim to replace powerful established tools like EIS or steady-state spectroscopy, but to offer an additional lens; one that is particularly suited for probing nonlinear, coupled, or emergent dynamics that define the real-world behavior of functional catalytic interfaces.

**Figure 1:**
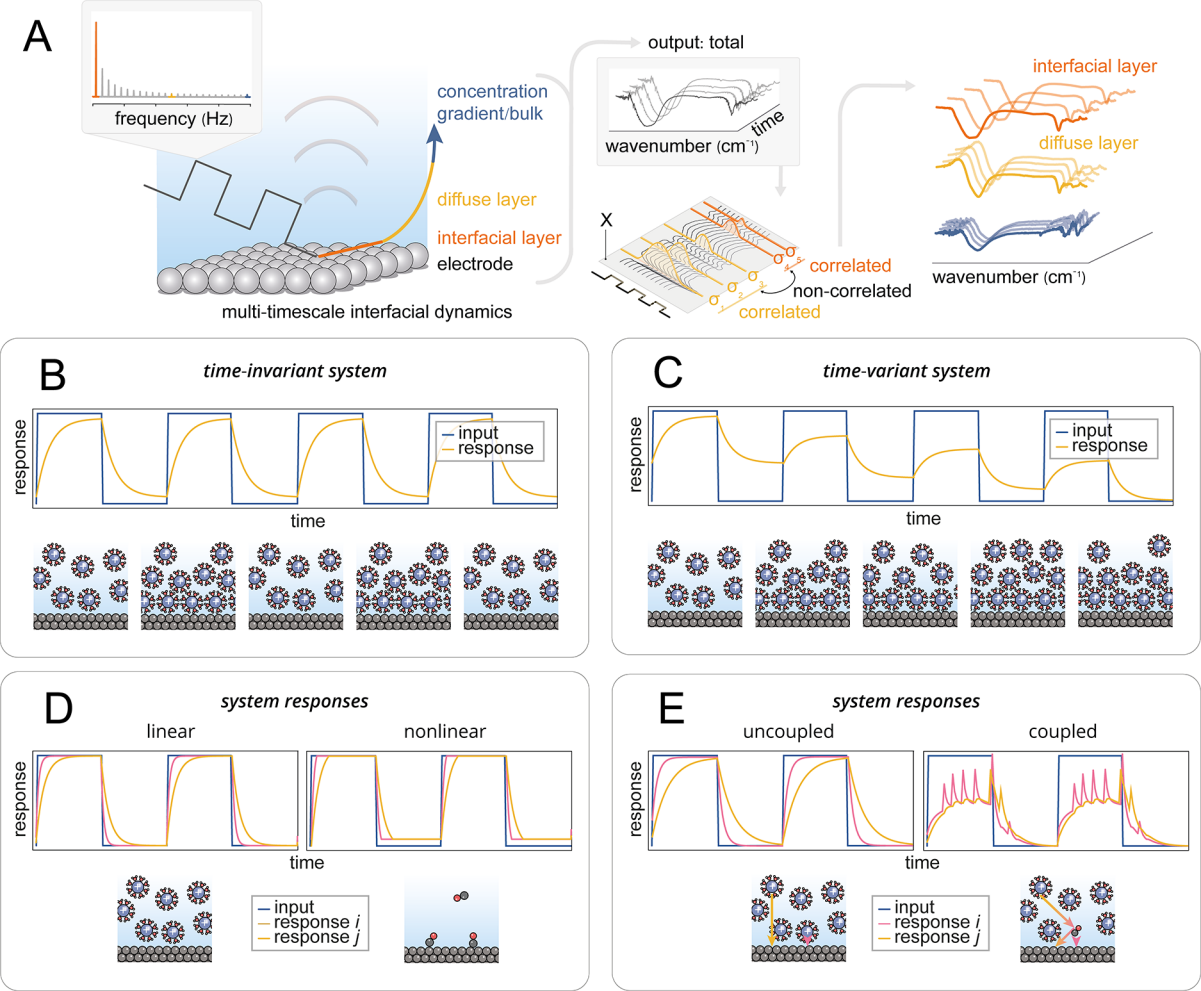
Foundational concepts underlying DRS, illustrating how different classes of system responses, linear vs nonlinear, time-invariant vs time-variant, and coupled vs uncoupled, emerge under structured electrochemical perturbation. (A) Overview of DRS, illustrating how temporally structured perturbations are used to probe (coupled) interfacial dynamics. (B,C) Illustration of system responses to pulsed electrochemical inputs in time-invariant (B) vs time-variant (C) regimes. In the time-invariant case, system parameters such as RC constants remain unchanged across pulses, yielding a consistent impulse response *h*(*t*). In contrast, for time-variant systems the RC characteristics evolve over time, for example due to cumulative cation buildup and incomplete discharge, so *h*(*t*) changes with each pulse and is dependent on the absolute time. The system does not fully return to its initial state, and the same input produces different outputs over time. (D) Comparison of linear vs nonlinear component responses. Linear responses (e.g., capacitive charging) exhibit proportionality between the input signal and system evolution, maintaining constant dynamics across amplitudes. Nonlinear responses (e.g., Langmuir-type CO adsorption) demonstrate amplitude- or state-dependent kinetics, including saturation, thresholding, or feedback. (E) Comparison of uncoupled vs coupled interfacial processes. In uncoupled systems, individual processes (e.g., interfacial vs diffuse layer charging) evolve independently, and the system response is the superposition of each component. In coupled systems, mutual dependencies, such as between CO adsorption and interfacial charging, lead to emergent behavior and dynamic interdependence between otherwise distinct components.

## Dynamic Response Spectroscopy

DRS provides a framework to interrogate interfaces beyond the constraints of linear, stationary, or reversible models. We reemphasize that although in the following we take electrochemical systems as the main example, the framework is equally applicable to catalytic interfaces in thermocatalysis and photocatalysis, where nonlinear restructuring, adsorbate dynamics, and feedback also govern performance.

Rather than assuming time-invariant behavior or weak perturbations, as done in classical approaches like EIS, we explicitly designed our experimental and data analysis pipeline to probe systems under real-world conditions, where responses are often nonlinear, history-dependent, and shaped by internal feedback. We do not intend to characterize systems at (assumed) equilibrium because (local) deviations are often, if not always, present. This is particularly true for electrocatalytic systems, which are by nature driven, nonequilibrium systems, where electrical potentials are applied, forcing, e.g., charge transfer, inducing ionic redistribution, or triggering surface transformations.^22,25^ A system’s departure from such assumed states is studied by resolving how spectral observables change in relation to structured perturbation, as visualized in Figure 1C, where the trajectory through which nonequilibrium conditions give rise to complex, time-evolving behavior enables mechanistic insights into coupled and time-variant processes that would otherwise remain obscured.

In DRS, we aim to disentangle the coupled dynamics of processes spanning multiple physical domains, such as charge transfer, catalyst degradation, ion transport, and interfacial restructuring. To observe, contrast is required, which in the DRS framework stems from the excitation of the different temporal response functions of the different processes at the interface. As outlined in Figure 1A, this is achieved by applying a structured stimulus, such as an electrochemical waveform (typically a square wave), with defined amplitude and position relative to the process of interest. Simultaneously, the system’s time-resolved response is recorded using spectroscopic techniques that capture a broad spectral range (nearly) simultaneously (e.g., broadband or white-light-based Fourier transform infrared spectroscopy, as opposed to step-scan IR). The resulting data matrices are then treated with multivariate decomposition techniques as multicomponent, time-dependent, potentially coupled, and not-necessarily periodic processes. The suitability of the applicable data analysis frameworks hinges on their ability to resolve and group the underlying physicochemical properties (via spectroscopy) and dynamical properties (component responses in time) without imposing equilibrium-based assumptions. In the following, we will discuss the formalization (supported with extensive mathematical explications and proofs in the Supporting Information Appendix) of the above heuristically discussed parameters to assess their boundary conditions and for deliberate experimental planning.

## Time (In)variance and Response Functions

Processes (or more precisely, distinct components at interfaces in catalytic systems) can exhibit characteristic time-dependent responses that reflect the underlying physicochemical mechanisms governing them.^22,26^ These responses often emerge from fundamental properties, which in electrochemistry may includeresistance (R) and capacitance (C), which together define a characteristic relaxation timescale known as the RC time constant, τ.^10^ This time constant describes, for example, the exponential charging behavior of a capacitor. In the context of interfacial electrochemistry, the electrode–electrolyte interface can often be conceptually partitioned into subregions, such as the compact (or interfacial) layer and the diffuse double layer (DDL).^27^ These layers are governed by different dielectric environments, ionic distributions, and degrees of molecular ordering, which, in turn, give rise to distinguishable response functions under external perturbation. When their dynamical timescales are sufficiently separated, these regions may be treated as quasi-independent contributors to the overall system behavior. To eventually interpret mechanistically how a time-dependent input *E*(*t*) gives rise to an observable spectroelectrochemical output *R*(*t*), we must first define the functional forms and physical interpretations of the underlying response functions *h*(*t*) that characterize each component, which will eventually allow us to dissect dynamic responses into separable and interpretable kinetic modes.

The simplest case of multicomponent response functions is the (linear) time-invariant (LTI) case, which is often approximated or imposed during measurements because of its simplicity. A system is time invariant when the response function *h*(*t*) is not dependent on absolute time (see also Supporting Information Appendix, Section S1A), or, in simple terms, when shifting the input signal in time results in the same shift of the output, with no change in the shape or characteristics of the response. Every input pulse yields the same output shape, fully returning to the baseline before the next.

As a simplified illustrative analogy, one might imagine the reorientation of water dipoles under an alternating electromagnetic field. When activated, these molecules are expected to reorient according to the direction of the field (with more layers reorienting as the penetration depth of the field increases, as illustrated in Figure 1B) only to return to an equivalent statistical equilibrium distribution upon deactivation. However, such linear time-invariant behavior is rarely, if ever, realized, especially under catalytic turnover, where nonlinearities are expected to emerge as a result of surface restructuring, solvent dynamics, and ion transport coupling across scales. Under these circumstances, larger magnitudes of the applied field may be nonlinearly screened by counterions in the double layer, and the reorientation of water molecules may not be reversible due to competitive adsorption at the catalytic surface or shifts in the balance of reaction intermediates (Figure 1C). But deviations from LTI are not merely mathematical curiosities. In catalysis, they can manifest as shifts in onset potentials, altered selectivity, or stability challenges under operando conditions.

Nevertheless, LTI provides a useful baseline to start discussing system dynamics. Figure 1B illustrates the idealized case schematically for an electrochemical RC-like interface where charging and discharging are symmetric and repeatable. Let us suppose our system has two components, say fast interfacial charging with τ_1_, and a slower response, perhaps diffuse-layer relaxation, with τ_2_. If the system behaves according to LTI assumptions, then understanding what each component is doing is straightforward, because under these conditions the system is predictable. If perturbed with one signal, a certain response is obtained; doing the same with two comparable signals at once gives the sum of those responses, which is what is known as the superposition principle.^5^ Mathematically, the system response can be expressed as a convolution integral

1 $$y\left(t\right)=\int_{0}^{t}h\left(t-\tau \right)x\left(\tau \right)d\tau$$

where *x*(τ) is the input (e.g., a square-wave modulation), *h*(*t* – τ) is the system’s impulse response function (which characterizes its memory), and *y*(*t*) is the output. Here, *h*(*t*) acts as a weighting function that emphasizes different portions of the input signal depending on the system’s internal dynamics. In LTI systems, *h*(*t*) remains fixed, meaning the system’s internal rules do not change with time. As such, and because the system responds proportionally to what is put in, reverse-engineering the system’s response functions (components) from the output is possible. Linear response theory is elegant, predictive, and often analytically tractable, enabling powerful analytical methods such as Fourier and Laplace transforms. This underlies the classical treatment of impedance, transient current decay, modulated excitation-phase sensitive detection, and so forth.^10,22,26^

However, real catalytic systems (particularly under catalytic turnover) rarely adhere to the assumptions of linear time invariance. In many cases, the system response function *h*(*t*) becomes time-dependent, meaning it no longer depends solely on the relative time difference (*t* – τ) but also on the absolute time *t*. This violates the conditions required for time invariance in convolution-based models. As discussed above, in an LTI system, if the input signal is shifted in time by some amount of τ, the output should shift identically, preserving its shape. That is, for an input *x*(*t*) and response *y*(*t*), time invariance implies: *x*(*t*) → *x*(*t* – τ) ⇒ *y*(*t*) → *y*(*t* – τ). This requires that the system’s impulse response *h*(*t*) is fixed over time, i.e., independent of when the input is applied. In contrast, time-variant systems exhibit a response function *h*(*t*) that changes over time. Even if the same input *x*(*t*) is applied at different times, differences in the environmental conditions of the system, such as local pH shifts or poisoning of the active sites, yield different outputs *y*(*t*) as a response. Mathematically, a simple illustrative example is *y*(*t*) = *t*·*x*(*t*), which is linear but not time-invariant, since the weighting (here, *h*(*t*) = *t*) changes with time. This behavior is schematically illustrated in Figure 1C, which depicts a system whose response evolves from pulse to pulse due to real-world factors. As a result, the system does not fully return to its initial conditions between perturbations, and the same input produces different outputs depending on the time it is applied.

Such time-variant behaviors are not edge cases but are widespread across catalysis and are precisely the type of dynamic complexity that DRS is designed to disentangle, with the aim of making these examples directly accessible to understand catalytic performance. Further examples include adsorbate-induced surface reconstruction, autocatalytic product accumulation, passive layer formation, and catalyst oxidation or carburization processes.^3,28,29^ For instance, in the electrocatalytic CO_2_ reduction reaction on copper, local pH changes occur as a function of reaction time,^30,31^ and electrodes are known to restructure.^32^ Additionally, the surface coverage of CO, a key reaction intermediate in this reaction, would eventually saturate via a Langmuir-type adsorption behavior. Such saturation behavior is an example of a nonlinear response, where the output does not scale linearly with the input, as illustrated in Figure 1D. Increasing the applied potential or reactant concentration does not linearly increase CO coverage once the surface is near saturation. Other common examples of nonlinear behavior in electrochemical systems include concentration-dependent ionic conductivity, field-enhanced adsorption or desorption, and non-Ohmic charge transfer kinetics, all of which are highly relevant in electrocatalysis.

In nonlinear systems, the convolution integral is no longer valid because the output at a given time depends not only on the current and past inputs but also on how these inputs alter the system’s internal state over time.^5^ Consequently, the superposition principle breaks down: the response to a combined input *x*_1_(*t*) + *x*_2_(*t*) does not (necessarily) equal the sum of the individual responses *y*_1_(*t*) + *y*_2_(*t*). This means we can no longer treat the system’s output as a linear combination of independently acting components and untangle them with standard linear tools.^33^ It is worth noting that Fourier analysis can still be applied to nonlinear systems in a formal sense (any signal can be decomposed into sinusoidal components), but interpreting the results becomes nontrivial. Nonlinearities often introduce new frequency content like harmonics, subharmonics or mixed-frequency terms that were not present in the input.^6^ These are not just (or only) artifacts; they can be fingerprints of the system’s internal complexity, but without a clear mapping between input and output, their physical interpretation, and distinguishing between artifact and meaningful event becomes ambiguous.

The final layer of complexity in the discussion of system responses, bringing us closest to realistic systems, is coupling. Coupling refers to the situation where the dynamics of one process directly influence or depend on the state of another, such as the activation of adsorbed CO_2_ molecules as the cations accumulate at an electrochemical interface during CO_2_ electroreduction.^21^ Importantly, coupling can be linear or nonlinear. In linearly coupled systems, the output remains a linear combination of the inputs, and the superposition principle still holds, for example, *y* = *a*_1_ *x*_1_ + *a*_2_ *x*_2_, where *x*_1_ and *x*_2_ are coupled but their interaction is additive. However, in nonlinearly coupled systems, one process modulates another in a state-dependent or multiplicative way, e.g., *y* = sin­(*x*_1_ + *x*_2_). Nonlinear coupling in electrocatalytic systems gives rise to emergent behavior that defies linear analysis. For instance, adsorbate coverage can modulate charge transfer kinetics, which in turn feed back into the interfacial potential or structure, producing history-dependent responses that cannot be decomposed into independent modes. In the case of CO_2_ reduction, we recently demonstrated that ion migration from the diffuse layer to the interface not only affects local conductivity or potential but actively modulates the CO_2_ to CO turnover rate,^21^ a process now also proposed as rate-determining in multiscale models.^34^ The double-layer structure, therefore, is not a passive background but a dynamic participant in catalysis. This underscores how DRS can directly link interfacial dynamical modes to catalytic turnover by providing a framework for connecting microscopic dynamics with macroscopic reactivity.

Such mutual interdependence between transport, adsorption, and reactivity prevents the clean separation of system components and gives rise to entangled dynamics. These can manifest as oscillations, bistability, or hysteresis, which are hallmarks of emergent behavior that lie beyond the scope of linear or decoupled models.^25^ Here, emergence refers to complex, system-level behavior that arises from interacting components, which is behavior that is not predictable from the parts alone.^35^ These features challenge reductionist assumptions and reflect a broader class of complexity found across physical, chemical, and biological systems.^35^ While Faradaic nonlinearities (i.e., those involving charge transfer) have received attention,^5,6,25^ there is no reason to assume that coupling or emergent dynamics are exclusive to them. Capturing these effects remains a major challenge and a central aim of the present framework.

## Input Functions Are Both Pillars and Tuning Knobs of DRS

The temporal structure and amplitude of the input perturbation directly define the kinetic modes accessible for observation^36–40^ and will define whether we have the possibility to disentangle the interplay of the hierarchy of relaxation processes, from picosecond-scale solvent reorientation near the interface,^41^ to millisecond-scale ionic relaxations in the diffuse layer, and second-scale bulk concentration gradients.^42,43^ These timescales are directly linked to catalytic function, as the relative rates of solvent reorientation, ionic relaxation, and diffusion can affect product selectivity and stability under operando conditions.

While sine waves are the canonical input for techniques such as EIS (10, 44, 45), their single-frequency nature under-samples the system dynamics (Supporting Information Appendix, Figure S8).^46–48^ In contrast, square and sawtooth waveforms (Figures S9–S10) provide multifrequency excitation, as these waveforms result from the sum of different frequencies, which can prove particularly effective at sampling system dynamics due to their sharp transitions and rich harmonic content (Figure 2A,B).^36,37,39,40,47^ Small perturbations (Δ*E* < ∼10 mV) probe the compact layer and fast interfacial processes, acting as a frequency filter and suppressing slow ionic modes.^10,45^ Larger amplitudes (Δ*E* ≳ 10–20 mV) drive the system into nonlinear regimes, accessing slow, non-Faradaic, and possibly Faradaic pathways (Figure 2C–E).^6^ However, we stress that no real interfacial catalytic system is strictly linear or time-invariant, even under these conditions, and purely linear behavior, when observed, is not a fundamental property but is either an approximation over a narrow perturbation window or simply a consequence of measurement technique or data analysis assumptions. If Δ*E* is much smaller than the thermal voltage (*kT*/*e* ≈ 25.7 mV), the ion distribution in the diffuse layer remains nearly Boltzmann-flat, and the potential drop is localized at the interfacial region. The capacitance of the diffuse layer, which scales as cosh­(*e*ψ/2*kT*) (where *e*, ψ, *k*, and *T* correspond to the elementary charge, electric potential at the interface, Boltzmann constant, and temperature, respectively) is approximately constant here, thus acting as a low-pass filter for high-frequency, low-amplitude stimuli.^10,44,45^ As such, small perturbations selectively probe fast, interfacial processes while filtering out slower, nonlinear ionic relaxations unless the amplitude is raised to activate them (see Figure 2C–E).

**Figure 2:**
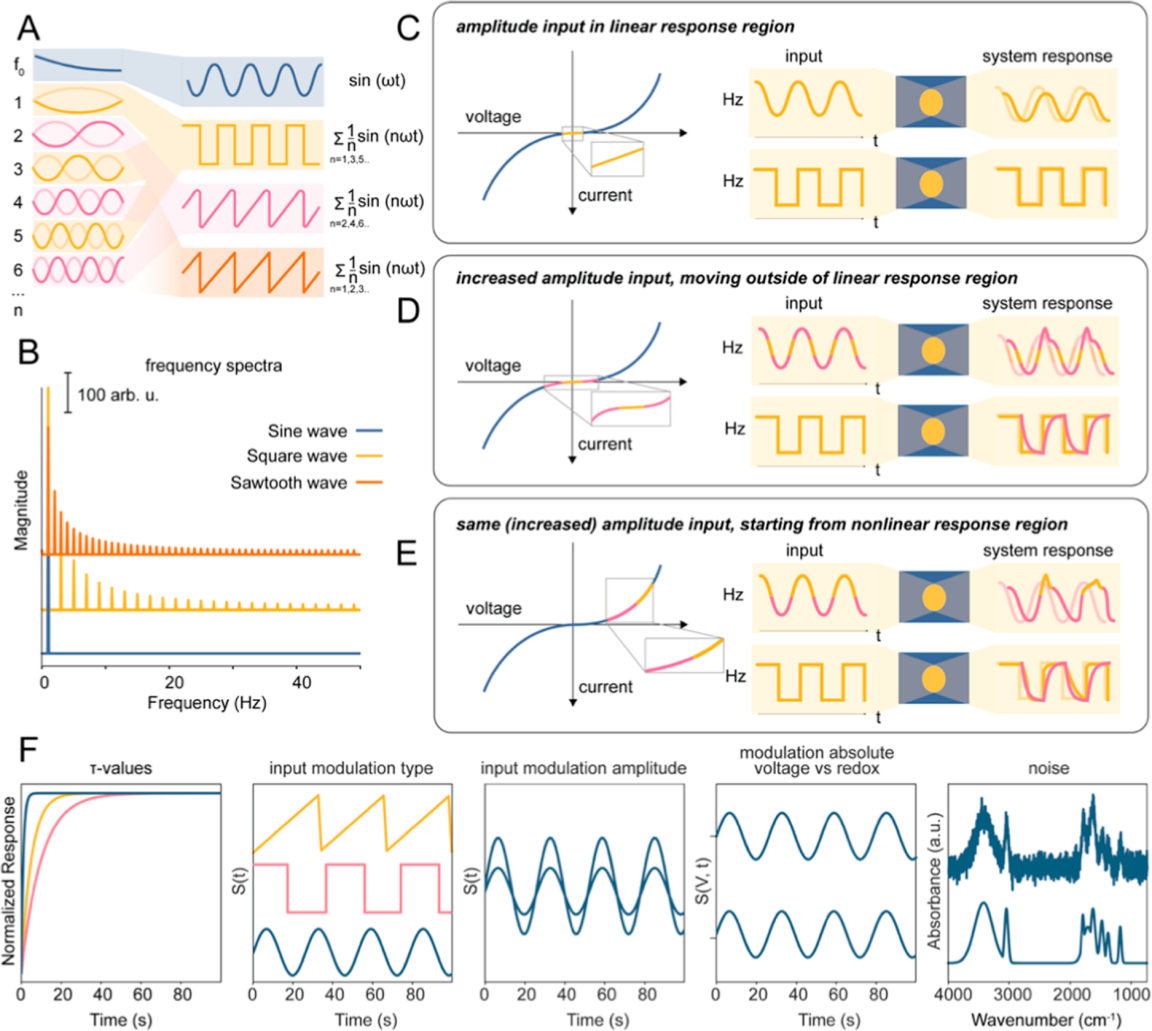
Key variables influencing the design of perturbation functions in DRS, including waveform composition, frequency content, amplitude regimes, and their impact on data interpretability and retrievability. (A) Summation of odd, and even, and all harmonics. (B) Frequency spectra of square, sine, and sawtooth waves. (C–E) Schematic illustration of the importance of picking an input function amplitude; staying in a linear regime increases the chances of adhering to superimposition principles in data analysis, while not doing so potentially increases information content in a single experiment, but not necessarily interpretability. (F) An overview of some of the parameters that influence retrievability of system characteristics, including the values of the characteristic time constants τ of each component (relative to each other), input modulation function, and amplitude, the absolute value of the input modulation relative to processes of interest (off/off, off/on, and on/on regimes), and noise in the spectral dimension.

The absolute value of the applied potential and its positioning relative to key system thresholds, e.g., onset of adsorption, redox, or point of zero charge, determine which components are active (“off/off”, “off/on”, “on/on”), each defining distinct observability regimes. To quantify how waveform and amplitude influence observability, we simulated synthetic electrochemical systems composed of dynamically independent or coupled components, spanning a wide range of time constants, amplitudes, and nonlinear interactions (Figure 2F). The governing model (eq S15; Supporting Information Appendix S2.1) includes linear and nonlinear couplings, component-specific kinetics, and perturbation shaping. We find that square waveforms maximize τ separation due to their transient-rich profiles, while sawtooth modulations better preserve the physical structure of response functions *R*_*i*_(*t*). The choice of input waveform thus imposes a trade-off between τ-resolution and fidelity of dynamic reconstruction (Supporting Information, Figures S8-S10, Table S1).

## Retrievability of Components, and Responses, and Disentanglement of Coupling

Disentangling characteristics of underlying system components from a single, convoluted scalar-valued measurement such as current is a classically ill-posed problem,^49^ let alone that it limits observables to that channel (i.e., Faradaic processes in this case). However, probing responses with broadband (i.e., spanning multiple wavenumbers), time-resolved, and chemically specific observables—such as ATR-SEIRAS, which probes several microns into the interfacial region, thus probing species spanning from the compact layer to the (near-)­bulk electrolyte^50^—greatly enhances separability by expanding the rank of the measurement space. The rest of the discussion is centered around (simulated) ATR-SEIRAS data, but the presented framework is, in principle, modality-agnostic, so long as time-domain resolution is preserved and not averaged out (i.e., excluding step-scan methods where temporal resolution is decoupled from spectral information). From classical signal processing literature, separability should be maximized when τ-ratios exceed 3–5 and spectral overlap is minimal, and this separability should degrade in the presence of coupling or noise.^51^ In catalytic systems, such degradation of separability is equivalent to losing mechanistic visibility of, for example, ion migration, solvent rearrangement, or adsorbate coverage as their changes may become conflated, which obscures their distinct roles in determining turnover.

Surprisingly, when using PCA to decompose a series of simulated spectral matrices spanning a wide range of parameters, which were analyzed for component retrievability (Supporting Information Appendix, Section S2, and Figure 3) we observe that systems with increasing τ-ratios (e.g., [1, 5, 10] or [1, 5, 25]) showed degraded performance in both τ-retrieval and dynamic reconstruction (*R*_*i*_(*t*) retrieval), especially under uniform amplitude conditions—contrary to classical signal processing expectations (Supporting Information Appendix, Tables S1–S2, Figures S8–S10). The likely explanation is that while increasing τ contrast might increase variance introduced by the spectral components, it also concentrates that variance into fewer principal axes, disproportionately amplifying slow-relaxing modes and suppressing faster dynamics. This biases PCA’s orthogonal basis away from a faithful recovery of all kinetic components (Supporting Information Appendix, Table S2).

**Figure 3:**
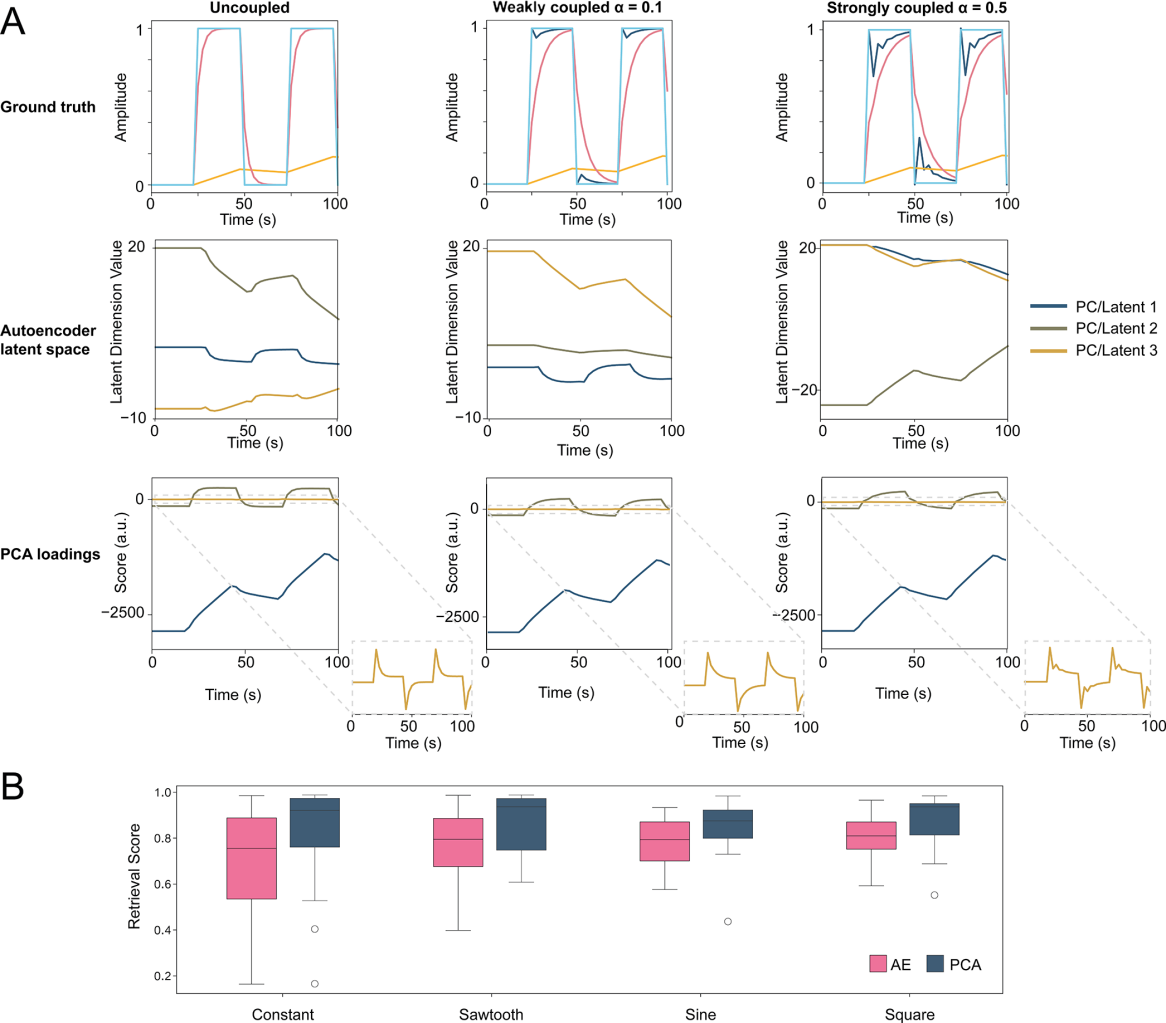
A comprehensive benchmarking of PCA and AE approaches for resolving dynamic components in spectroelectrochemical systems, illustrating how coupling strength, modulation waveform, nonlinearity, and spectral structure influence the retrievability of kinetic information. (A) PCA decomposition of synthetic spectral datasets composed of three kinetic components: a fast linear term, a moderate exponential decay, and a nonexponential delayed onset. Left to right panels show decompositions for systems with no coupling (α = 0), weak coupling (α = 0.1), and strong coupling (α = 0.5). Response functions or ground truths are given in the top row, AE latent dimensions as a function of time in the middle row, and PCA loadings in the bottom row. (B) Boxplot comparing retrievability across different waveform inputs (sawtooth, sine, square, constant). While all modulations produce high median scores, sawtooth and square modulations offer slightly improved retrieval performance compared to sine or constant inputs, likely due to their smoother driving dynamics. Figures S12–S14 give a detailed overview of all benchmarking.

Through this analysis, we find that *R*_*i*_(*t*) retrieval was highest when τ values were similar and amplitudes were nonuniform, as this suppressed dominance of any single component and allowed more equitable variance partitioning. Furthermore, we find that while coupling may be present in the system, it may be embedded until sufficiently strong; Figure 3A shows PCA and AE latent space representations of spectral matrices with varying coupling strength (parametrized by the coupling strength α = 0, 0.1, and 0.5), and as also shown in Figures S12 and S13 for more continuous variable space representation. PCA decompositions of the uncoupled and weakly coupled datasets (α = 0, 0.1) yield nearly identical scores and loadings (a measure of the underlying dynamics and the spectral features associated with them, respectively), showing that component identity and kinetics are retrieved with high fidelity for uncoupled and weakly coupled systems, with embedded but not visible coupling for the latter. The dominant axes of variance remain aligned with the original input processes, indicating that low-amplitude coupling does not significantly perturb the statistical structure of the dataset, which is another demonstration of the conceptual suitability of PCA to separate processes based on their time variance. However, at stronger coupling (α = 0.5), clear deformation of the PCA score trajectories and loadings emerges. Temporal signatures of the coupling term begin to bleed into the principal components, indicating that the interaction strength has crossed a detectability threshold in the PCA embedding space. This observation defines a boundary in observability space: below a critical level of interaction-induced variance or temporal contrast, PCA fails to register coupling. Beyond that threshold, the covariance structure of the dataset is sufficiently perturbed to reflect dynamical entanglement in its dominant components, and while similar trends are observed for AE, both component retrievability and coupling seem to be showing less strong trends. In fact, while nonlinear dimensionality reduction techniques such as AEs are theoretically well-suited for capturing time-evolving, spectrally entangled responses without assuming linearity or orthogonality, our systematic simulations reveal a counterintuitive result.

The comparison of PCA and AE across a broad sweep of nonlinear and coupled systems (Figures S12 and S14) reveals a robust, and initially surprising, trend which is that PCA often outperforms AE in retrieving physical response functions, even under nonlinear conditions. This result holds across variation in waveform, spectral overlap, coupling strength, nonlinear interaction terms (β), noise, and activation states. While AEs capture nonlinear manifold structure and outperform PCA in certain high-noise regimes, their latent axes do not consistently align with physically interpretable kinetics. Instead, PCA (despite its constraint of orthogonality) remains better at retrieving *R*_*i*_(*t*) structures when responses are sufficiently separated in time. We attribute this to the fact that PCA prioritizes directions of maximal variance, which in these systems correlates strongly with relaxation hierarchy and kinetic contrast. Thus, even when AE provides a compact latent embedding, it does not guarantee interpretability. This highlights a broader methodological point, which is that model-lean representations must be evaluated not only by compression or reconstruction error, but by their alignment with physically meaningful observables, when possible. Indeed, it shows that for AE, systems- (physics-)­informed design of information analysis pipelines^52^ or the use of probabilistic generative models such as variational autoencoders^53^ are likely necessary and can potentially further aid in the retrieval and disentanglement of system parameters like coupling type, degree, and so forth. This deserves further investigation but is beyond the scope of the present work. For catalysis, these benchmarking results emphasize that even simple dimensionality reduction methods, if carefully applied, can provide interpretable mechanistic insights into interfacial dynamics that directly impact activity and selectivity.

## Practical Considerations

Realistic electrochemical systems can involve far more components and coupling pathways than the scenarios discussed above. As such, the full utility of the separability-retrievability framework can only be realized through a carefully designed and executed series of experiments. A robust DRS study must align operando reactor design, the perturbation protocol, detection scheme, and data analysis method (Figure 4A) with the physicochemical characteristics of the system and the specific hypothesis under investigation (e.g., to study the composition of the double layer, to gain mechanistic insights, study coupling, and so forth). These choices must be statistically rigorous and reproducible.

**Figure 4:**
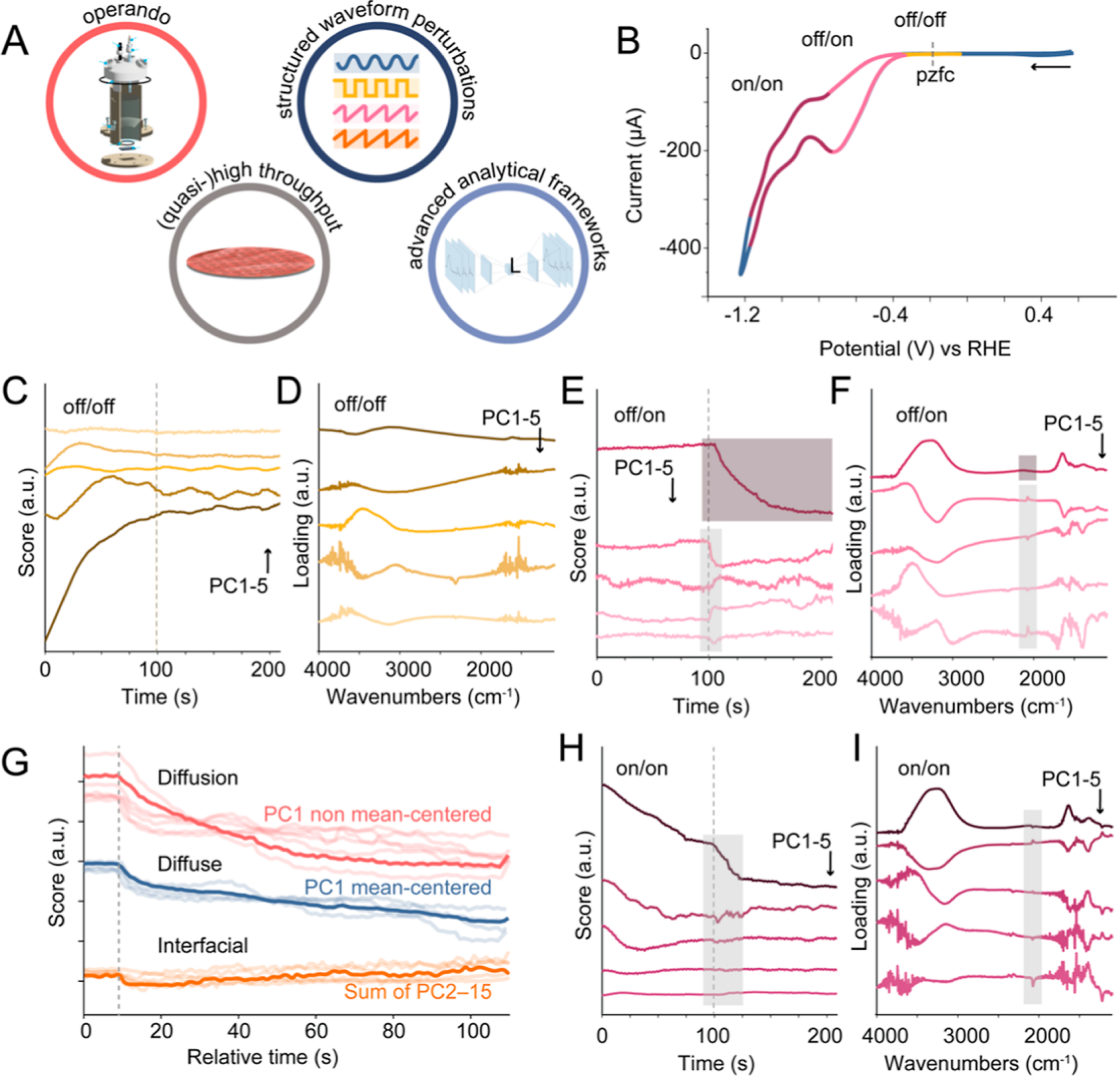
Application of DRS to experimental ATR-SEIRAS data reveals how interfacial dynamics during CO_2_ electroreduction on copper evolve across distinct potential regimes, highlighting the impact of activation states on spectral decomposability. (A) Important principles for the application of DRS: operando measurements in reactors operating as close to realistic systems as possible, reproducibility via (quasi-)­high throughput experimentation, structured waveform perturbations, and advanced data analysis frameworks. (B) Cyclic voltammogram of CO_2_ on a polycrystalline copper electrode using a 0.2 M NaHCO_3_ electrolyte, which allows for the determination of three regimes: −0.05 to −0.4 V_RHE_ (off/off, yellow), −0.4 to −0.8 V_RHE_ (off/on, pink), and −0.8 to −1.1 V_RHE_ (on/on, dark red). In each of these regimes, a pulsed potential experiment was performed, and characterized by ATR-SEIRAS (see Figure S12 for full spectral matrices, and electrochemical data); (C–F,H,I) PCA decomposition of these pulsed potential ATR-SEIRAS experiments in the three different regimes (off/off, off/on, on/on). The loadings and scores, respectively, of the first 5 PCs are given in (C,D) for −0.05 to −0.4 V_RHE_ (off/off, yellow), (E,F) −0.4 to −0.8 V_RHE_ (off/on, pink), and (H,I) −0.8 to −1.1 V_RHE_ (on/on, dark red). (G) The three groups of score profiles consistently apparent from PCA decomposition of the −0.4 to −0.8 V_RHE_ experiment shown in (E,F); obtained by averaging the response of PC 1 of the nonmean-centered data, and PC 1, and PC 2–15 of the mean centered data.

Unlike the simulations presented here, where ground-truth response functions are known, experimental systems operate under physical constraints that limit the direct access to such truths. Nevertheless, retrievability can still be evaluated: either through adherence to established physical laws (e.g., diffusion scaling) or through consistency across a series of experiments. For instance, although we cannot necessarily directly a priori fit component-wise response functions to known models, we can assess whether retrieved dynamics, like exponential decays or charge–discharge asymmetries, behave in ways that are physically and chemically consistent.

To contextualize the framework, we examine representative experimental use cases of the electrocatalytic CO_2_ reduction reaction (CO_2_RR) on polycrystalline Cu in 0.2 M NaHCO_3_, studied by ATR-SEIRAS (details in Materials and Methods, and the Supporting Information, Section S3, additional details can be found in ref 21). In this system, we expect at least two dominant dynamical components; a fast interfacial layer (IL), where adsorbates like *CO accumulate (peak at ∼2070 cm^–1^), and a slower DDL, characterized by structured water modes (e.g., ∼3210 cm^–1^). The fidelity of retrieving τ_DDL_ can be evaluated by comparison with known diffusion coefficients or concentration-dependent trends. CO_2_RR in this case serves as an ideal test case because of the clearly resolved IR peak of *CO, which can act as a marker for interfacial species. A successful decomposition would yield separate principal components for the IL and DDL, each with distinct spectral features and temporal dynamics. To illustrate how these concepts connect with the simulated separability map in Figure 3 and S12–S14, we present in Figure 4 a set of square-wave pulsed potential experiments probing three potential windows identified from the cyclic voltammogram presented in Figure 4B: off/off (−0.05 ↔ −0.4 V_RHE_), off/on (−0.4 ↔ −0.8 V_RHE_), and on/on (−0.8 ↔ −1.1 V_RHE_). As shown in Figure 4C–H, only the off/on regime yields principal components that clearly separate IL and DDL dynamics. Specifically, *CO modes appear in the loadings of higher-order PCs with fast, linear decays in their scores, while water-related features dominate PC1 with slower exponential relaxation (Figure 4E,F). In contrast, in the on/on regime (Figure 4G,H), spectral and temporal features are entangled, *CO appears across multiple PCs, and no clean separation of dynamical modes is achieved, which suggests insufficient decoupling of interfacial and diffuse layer responses under our employed experimental constraints. That is, under current time resolution (∼1.1 s) we are prohibited from resolving the true charging and relaxation dynamics of the DDL, where faster acquisition (e.g., in the μs range) may enable the recovery of fast interfacial processes even in the absence of Faradaic turnover. Moreover, although we treat the IL as a single functional layer, in reality it likely hosts a hierarchy of overlapping species whose responses are only partially separable, underscoring the value of improving high-dimensional decomposition methods as well as experimental approaches.

Additional evidence for the physical validity of these separations is detailed in prior work,^21^ where exponential decay rates of the DDL components tracked the expected trends with changing electrolyte concentration, and their asymmetry across pulse cycles reflected known cycling behavior. These findings collectively demonstrate how retrievability and separability, even in experimental settings, can be inferred through internal consistency, known chemical behavior, and well-chosen spectral markers, even without access to the exact ground truth.

To further clarify and preempt possible misconceptions, we emphasize that the dynamical modes we label as “interfacial,” “diffuse,” and “diffusion” do not map directly onto classical EDL constructs such as known from the Gouy–Chapman–Stern model.^54^ Those models, based on dilute solution theory, linear response, and purely electrostatic equilibration, typically predict submillisecond relaxation times. In contrast, what we observe are emergent, system-level responses that couple charge rearrangement, ion correlations, solvent structure, and interfacial speciation. Figure 4G exemplifies this; the principal component score profiles extracted from the off/on regime (Figure 4E,F) consistently exhibit three dominant (groups of) timescales. The fastest response (<1.1 s) likely reflects capacitive charging and adsorbate reorganization, though even here, chemical steps such as hydration shell collapse may intervene.^55,56^ The intermediate response (∼10 s) bears signatures of correlated ion rearrangements or overscreened DDL regions, whose relaxation clearly deviates strongly from textbook Poisson–Boltzmann behavior. The slowest component (>100 s) cannot be captured at all by classical EDL theory. It likely involves proton buffer dynamics, bulk ion redistribution, and solvent network reorganization such as expected in a diffusion layer or concentration gradient. These processes are not temporally fully orthogonal due to their shared coupling via the electric field, solvation shell dynamics, and local reactivity, but they are separable by variance under these conditions in the sense that DRS can isolate their dominant timescales and spectral fingerprints. In this way, DRS allows us to go beyond resolving theoretical layers, by revealing functionally distinct regimes of interfacial dynamics, each arising from the subtle interplay between charge, structure, and chemical environment. The result is a dynamic map of the evolving complexity of the double layer and beyond, retrieved not by assuming classical behavior, but by letting the system reveal its own dominant temporal modes.

## Outlook

At heart, the framework proposed is ultimately aimed at helping refine and, where appropriate, revise the physical models that underlie our understanding of catalytic interfaces. This work presented the case of electrocatalytic systems, including those related to transport, double-layer structure, and dynamic coupling. When applied systematically, DRS offers a means to delineate the regimes in which existing models hold across catalysis and those where they fail to describe observed behavior. In doing so, it may provide a route not only to sharpen existing theories but also to uncover previously hidden dynamics and emergent interfacial phenomena.

Pursuing this level of mechanistic insight will place increasing demands on analytical strategies. Not only in experimental design but, perhaps more critically, in how we decompose and interpret time-resolved data. While PCA remains a mathematically rigorous and model-free method for dimensionality reduction, it excels in systems where the sources of variance are globally expressed (i.e., present throughout the dataset) and uncorrelated. Under these conditions, PCA constructs an orthogonal basis that reflects the intrinsic structure. However, in complex, dynamically coupled systems, PCA decompositions can become difficult to interpret, misrepresent component dynamics, or fail to isolate them entirely. In our own simulations, weakly coupled dynamics are often entirely missed. Even when they do dominate variance, interpretation can become obscured when physical sources overlap or evolve over time. Related methods such as independent component analysis (ICA) which target statistical independence rather than orthogonality are also limited in applicability when physical processes are deterministically coupled or statistically dependent. These limitations underscore the need for decomposition approaches that do not rely solely on global linear assumptions but are instead guided by the known physical topology and symmetry of the system, including known constraints, conservation laws, and anticipated sources of dynamical variation. For example, dynamic mode decomposition (DMD) offers a way to extract temporally coherent structures from time-resolved data, but especially so when system responses are governed by recurrent or modal behavior (requiring periodicity, LTI), and some knowledge of predetermined temporal behavior.^5,6,57^ Non-negative matrix factorization (NNMF), or multivariate curve resolution alternating least-squares (MCR-ALS) may aid in reproducing component spectra when signals are additive but not necessarily orthogonal. However, these approaches rely on positivity and closure constraints that are not always appropriate for the experiments proposed, particularly when using infrared spectroscopies like ATR-SEIRAS, where negative absorbance differences and nonconserved species (e.g., concentrations) are common.

A more promising route lies in emerging tools from machine learning and nonlinear dynamics. Kernel PCA expands the reach of classical PCA by embedding the data in a higher-dimensional feature space, effectively linearizing nonlinear relationships. Autoencoders, a class of neural network architectures designed to compress (encode) and reconstruct (decode) high-dimensional data, can identify latent coordinates that capture system behaviors theoretically even under strong nonlinear coupling.^53^ We explored their use here and found that even simple implementations can retrieve relevant low-dimensional structure, though they are not a universal solution, and underperform against PCA. Incorporating prior knowledge, e.g., through physics-informed AEs,^52,58,59^ or recurrent neural network (RNN)-based architectures aimed at to processing sequential data,^60^ may offer greater power and interpretability, especially when applied to time-resolved data where latent coordinates correspond to dynamic physical phenomena such as adsorption, restructuring, or phase changes.^61^ There is rich literature on disentangling latent space coupled variables and on interpretability of latent space such as, e.g., traversing.^53,62–66^ The marriage of such architectures informed by simulations, for example, ab initio molecular dynamics, with architectures informed by experimental results offers a particularly exciting pathway for exploration of cross-modal improvement in models, interpretability, and so forth. Crucially, the use of these advanced tools does not come at the cost of physical interpretability. When grounded in appropriate constraints and trained with rigor, they offer a means to align the complexity of the analysis method with the complexity of the system under study.

In this light, DRS becomes more than a probe of kinetics or mechanisms; it becomes a test of observability itself. What aspects of system behavior can be reliably resolved and which remain hidden is ultimately dictated by the compatibility between physical complexity and the analytical lens applied. As scientists, we continually aim to calibrate our understanding, not only through our models but also through the methods we employ. Ideally, these two domains, model and method, should coevolve, negotiating the boundary between what is measurable and what is meaningful. With the analytical and computational tools now at our disposal, we are entering an era where such integration is not only possible but also increasingly necessary. The framework presented here offers a pathway into this space. However, the deeper challenge remains and must be noted; to recognize the blind spots intrinsic to any tool and to strive for an account of physical reality that is both rigorous and honest. That is, we must remain attentive to how our frameworks shape what we are able to perceive and, just as importantly, to what they may prevent us from seeing.

## Concluding Remarks

This work details and examines a practical *emergentist* framework for probing complex dynamic, multiscale behavior at catalytic interfaces through time-structured perturbations and spectroscopic detection. We demonstrate that even unsupervised linear decomposition methods like PCA can extract mechanistically meaningful dynamic features, provided the experiment is carefully designed, rigorously employed across a series of variables, and the limits of each analytical method are understood. At the same time, we show that PCA may obscure or entirely miss parts of overall system dynamics when variance is locally expressed or weakly coupled. DRS is not a singular method, but a modular approach, one that links perturbation design and data decomposition to the structure and dynamics of the system under study. Its strength lies in this integration, and by coordinating timescales of stimulation and detection, DRS enables the physicochemical separation of interfacial electrochemical processes, whether Faradaic or not. The future of this approach likely lies in deliberate experiment–model alignment. That is, using advanced decomposition tools where necessary, or helpful, but always grounded in physical understanding and statistical rigor. The goal is not abstraction, but clarity; to resolve not only what is present at the interface, but how it evolves and interacts. As our systems grow in complexity, so too must our frameworks, not for the sake of complexity itself, but to remain faithful to the complexity of the interfaces we seek to better understand. This perspective thus highlights how DRS can serve as a practical tool for catalysis research, enabling operando access to the dynamic couplings that ultimately govern interfacial catalytic function.

## Materials and Methods

Full details on the employed mathematical derivations, models, and methods can be found in the Supporting Information Appendix. In brief, we introduce the mathematical foundations of the framework, where we define and analyze the effects of (non)­linearity and the degree of coupling in data disentanglement and retrievability by a multivariate simulation framework, where we calculate the responses of different spectral components to multiple perturbation functions with different variables like decay rates, and considering several degrees and types of linear and nonlinear coupling among the components (see Section S2 in the Supporting Information). The analysis of the simulated results was performed using both PCA, via sklearn.decomposition.PCA from the scikit-learn 1.6.1 library, and autoencoders (and PSD and ICA in the Supporting Information Appendix, for comparison). For the analyses of the latent features using autoencoders, a feed-forward autoencoder using PyTorch 2.7.0 was implemented. The encoder consisted of two fully connected layers (nn.Linear) with ReLu activation (nn.ReLu): an input layer matching the spectral dimension, a hidden layer with 64 neurons and a ReLu activation, and a bottleneck layer with three latent dimensions. The decoder mirrored this architecture in reverse using nn.Sequential containers. The autoencoder was trained using the Adam optimizer (torch.optim.Adam) with a learning rate 1 ·10^–3^ and a mean-squared error loss (nn.MSELoss). Training proceeded for 30 epochs with a batch size of 4. Estimations of τ_*i*_ were performed by fitting through scipy.optimize.curve_fit (1.15.2) exponential rise functions to the time-resolved latent trace using SciPy 1.15.2 (scipy.optimize.curve_fit). All data analysis was performed in Python 3.11.12 using a self-written code. Visualization was performed using matplotlib 3.10.1 and seaborn 0.13.2.

## Supplementary Material



## Data Availability

All data used in this publication (upon publication) are shared in an online repository and are available for public use. All Python scripts employed can be found (upon publication) in the Vogt Lab Github account, available at https://github.com/VogtLab.

## Notes

Additional discussions on the mathematical framework of DRS; analysis on the separability of simulated time series under different degrees of linearity and coupling using PSD, principal component analysis and autoencoders; experimental time-resolved ATR-SEIRAS spectra from 0.2 M NaHCO_3_ solutions while performing DRS to disentangle the CO_2_RR under different potential windows; and eigen decomposition plots of the same datasets (PDF)
