# Supplementary material for: Dynamic Response Spectroscopy: An Emergentist Framework for Multi-Timescale Catalytic Interfacial Dynamics
Source: ACS Catal. 2025 Nov 7;15(22):19397–409. doi: 10.1021/acscatal.5c05171 (PMC12645478; doi:10.1021/acscatal.5c05171)
Supplement: Supplementary file 1 [file cs5c05171_si_001.pdf]

Supporting Information for:

Dynamic Response Spectroscopy: an Emergentist Framework for Multi-Timescale Catalytic Interfacial Dynamics

Daniel Sinausia[1], Florian Meirer[2], Anatoly I. Frenkel[3, 4], Charlotte Vogt[1]*

[1] Schulich Faculty of Chemistry and Resnick Sustainability Center for Catalysis, Technion - Israel Institute of Technology, 3200002 Haifa, Israel.

[2] Inorganic Chemistry and Catalysis, Institute for Sustainable and Circular Chemistry, Utrecht University, 3584 CG, Utrecht, The Netherlands

[3] Department of Materials Science and Chemical Engineering, Stony Brook University, 11794, Stony Brook, New York, United States

[4] Division of Chemistry, Brookhaven National Laboratory, 11973, Upton, New York, United States

* **Email:** c.vogt@technion.ac.il

Table of Contents

1. Mathematical Framework ... 3
  1.1 Response Functions ... 3
    1.1.1 Linear Response and Convolution ... 3
    1.1.2 Beyond Linearity: When Systems Misbehave ... 4
  1.2 Input Functions ... 4
  1.3 Complementary Observables ... 5
  1.4 Separability from Classical Signal Processing Literature ... 6
2. Retrievability and Disentanglement of Coupling ... 7
  2.1 Simulation Framework ... 7
  2.2 Disentangling Responses ... 8
    2.2.1 Phase Sensitive Detection ... 9
    2.2.2 Principal Component Analysis ... 11
    2.2.3 Autoencoder ... 12
  2.3 Separability Criteria ... 14
    2.3.1 Linear and Uncoupled Systems ... 14
    2.3.2 Nonlinear and Coupled Systems ... 15
  2.4 Test Cases ... 16
    2.4.1 Uncoupled Linear Systems ... 16
    2.4.2 Nonlinear and Coupled systems ... 21
3. Supplementary Experimental Results ... 26
4. Supplementary References ... 28

1. Mathematical Framework

1.1 Response Functions

To understand how and under what conditions a time-dependent input E(t) gives rise to a measurable spectroelectrochemical output R(t) we begin with linear time-invariant (LTI) systems.

1.1.1 Linear Response and Convolution

In the regime of *linear time-invariant (LTI)* systems, the relationship between an input and its corresponding output is fully characterized by the impulse response function, h(t), which describes how the system responds to an instantaneous perturbation, a Dirac delta function δ(t). For an arbitrary input E(t), the output R(t) is given by the convolution of E(t) with h(t):

$$R(t) = \int_0^t h(\tau)E(t-\tau)d\tau \qquad [1]$$

Here, τ is the dummy variable of integration, representing time since the input event. For causal systems, as is typical to electrochemistry where the response would not precede the input, the system response is defined only for t ≥ 0, and is multiplied by a Heaviside step function Θ(t), ensuring that h(t) = 0 for t < 0. As a simple and illustrative example, consider a first-order process such as an RC charging circuit or capacitive double-layer charging. The impulse response is:

$$h(t) = \frac{1}{\tau}e^{-\frac{t}{\tau}}\Theta(t) \qquad [2]$$

where τ is the system's characteristic relaxation time (e.g., RC time constant). For a step input $E(t) = E_0\Theta(t)$, the response becomes:

$$R(t) = E_0\left[1 - e^{-\frac{t}{\tau}}\right] \qquad [3]$$

This describes the canonical exponential rise to steady state observed in capacitive charging or in time-resolved spectroscopic signals from interfacial species. Real systems, however, rarely consist of a single time constant. In practice, multiple independent processes contribute, each with their own relaxation behavior. If we consider two independent first-order processes, one fast (τ_1), and one slow (τ_2), the total impulse response becomes:

$$h(t) = A\frac{1}{\tau_1}e^{-\frac{t}{\tau_1}} + B\frac{1}{\tau_2}e^{-\frac{t}{\tau_2}} \qquad [4]$$

and the corresponding response, output, to a step input is:

$$R(t) = E_0\left[A\left(1 - e^{-\frac{t}{\tau_1}}\right) + B\left(1 - e^{-\frac{t}{\tau_2}}\right)\right] \qquad [5]$$

The result is a two-phase rise, with distinguishable fast and slow dynamics, each term corresponding to a mechanistically distinct process. In electrocatalytic contexts, this could map onto adsorbate adsorption and interfacial layer charging (fast), and ion diffusion in the diffuse double layer (slower). Classic transient electrochemical analysis often makes this decomposition explicit (1, 2). For example, the current following a potential step is often modeled as:

$$i(t) = i_{dl}(t) + i_{Faradaic}(t) \qquad [6]$$

Where $i_{dl}(t) = C\frac{dE}{dt}e^{-t/\tau_{RC}}$ represents capacitive decay, and $i_{Faradaic}(t) \propto t^{-1/2}$ describes the Cottrell response of diffusion-limited electron transfer. These responses are distinct in both form and timescale, enabling their separation and parameter extraction.

In the Dynamic Response Spectroscopy (DRS) approach, these principles extend from scalar observables (e.g., current) to time-resolved spectroscopic signals. At each wavenumber p, the observed spectral intensity $S_p(t)$ is modeled as the sum of distinct dynamical components:

$$S_p(t) = \sum_j S_{p,j}^{(proc_j)}(t) \quad [7]$$

Each term corresponds to a process $proc_j$, such as adsorption, reorganization, or diffusion, each of which may follow exponential, sigmoidal, or power-law dynamics depending on the mechanistic origin.

1.1.2 Beyond Linearity: When Systems Misbehave

Response theory is elegant, predictive, and often analytically tractable. However, real electrochemical interfaces rarely conform to such simplicity. They restructure, couple, and saturate. They evolve in ways that depend on both the current state and the history of perturbation. In these cases, the assumptions of linearity and time-invariance break down, along with the convenience of convolution, and we enter the domain of nonlinear, state-dependent behavior. Below, we formalize two representative deviations from the ideal LTI.

Case 1: Nonlinear but Uncoupled Systems

In this class of systems, multiple physical processes operate independently, but at least one exhibits a nonlinear dependence on the input. A classic example is a saturation behavior (3), such as:

$$R(t) \propto \frac{E(t)}{1+E(t)} \quad [8]$$

Here, even though each individual process follows its own characteristic timescale and evolves independently of the others, the superposition principle fails as the response to $E_1(t) + E_2(t)$ is not the sum of the individual responses. The impulse response h(t) becomes input-dependent, and the system becomes a nonlinear time-invariant (NLTI) system, invariant in time (i.e., the manner in which the system responds to an external stimulus does not change over time), but nonlinear in how input maps over time to output (4).

Case 2: Nonlinear and Coupled Systems

The most complex (and arguably most realistic) scenario involves nonlinearity coupled with inter-process interaction. In such systems, the dynamics of one process influence, and are influenced by, the state of another. Consider, for example, the coupling between diffuse layer charging and interfacial adsorption where the local electric field arising from the charged double layer may alter the solvation shell or adsorption energy of a surface-bound intermediate (5, 6). Such a system can be described by coupled nonlinear differential equations:

$$\frac{dR_1}{dt} = f_1(E(t), R_2(t)), \frac{dR_2}{dt} = f_2(E(t), R_1(t)) \quad [9]$$

Here, the evolution of R_1 and R_2 (e.g., corresponding to diffuse and interfacial layer responses) are explicitly interdependent. This class of systems is generally nonlinear and time-variant, i.e., the mapping from input to output changes with system state, and often with time, for example due to restructuring, aging, or other history-dependent transformations.

1.2 Input Functions

Input functions in the discussed approach are used to create measurable contrast; differentiation in the to-be-probed processes which in this case is based mainly on the contrast of time (response functions). Just as the probe in for example optical microscopy defines the resolution and contrast of the observed transitions, so too does the shape of the electrochemical input govern which interfacial dynamics are excited, amplified, or obscured (7–11). The sine wave remains the

canonical input in electrochemical systems, as exemplified by EIS, wherein a single frequency component allows the response of each linear element to be measured independently, yielding a straightforward association between phase lag and time constant (12–14). Different waveforms can be represented in terms of their harmonic content by Fourier series, the Fourier decomposition of a square wave, for example:

$$S(t) = \sum_{n=1,3,5,\ldots}^{\infty} \frac{4}{\pi n} \sin(n\omega t) \quad [10]$$

contains only odd harmonics, but with amplitudes that decay slowly (∝ 1/n), maintaining significant energy across both low and high frequencies (Figure 2A, B). This allows the simultaneous excitation of processes with widely spaced time constants. By contrast, sawtooth waves, comprising both even and odd harmonics:

$$S(t) = \sum_{n=1}^{\infty} \frac{2}{\pi n} (-1)^{n+1} \sin(n\omega t) \quad [11]$$

feature steeper harmonic decay, which can limit their ability to drive fast interfacial modes, but their amplitudes decay more steeply, limiting excitation of high-frequency processes. There are many excellent literature examples on the use of multi-frequency perturbations, including square waves, sawtooth waves, multisine signals, and multi-step ramps for electrochemical interrogation (7, 8, 10, 11, 15).

1.3 Complementary Observables

No single observable can fully characterize the internal state of a complex dynamical system. In systems theory, a system is said to be observable if its internal state can be reconstructed from its outputs (16). If certain internal dynamics do not measurably influence the recorded signal, they remain hidden, regardless of how sensitive the detector may be. To consider an example, the common electrochemical observable: current. A measured current signal captures only the net transfer of electrons and capacitive charging. Structural reorganization, field-modulated solvation, or changes in adsorbate conformation, phenomena that can be central to reactivity, may occur without producing a measurable faradaic or capacitive response. For example, the potential-induced reorientation of interfacial water molecules, which modulates local reaction environments through solvation effects, can proceed without net charge transfer and is thus electrochemically silent. Without a complementary measurement channel sensitive to these dynamics, such as time-resolved vibrational spectroscopy, such processes remain inaccessible. This concept of measurement bottlenecks is well-formalized in information theory (17, 18). Each measurement channel offers a finite information capacity. Even the most carefully designed waveform, applied to a single observable, cannot fully span the multidimensional space of coupled interfacial dynamics. Thus, expanding the number and nature of observables, particularly those that project onto quasi-orthogonal, i.e., weakly correlated physical processes, substantially enhances the effective rank of the measurement matrix. In doing so, one moves from an underdetermined inverse problem to a better-posed reconstruction of latent physical processes. We discuss this more formally below.

The resulting current transient to a voltage step is often modeled as a sum of exponential decays, each associated with a distinct relaxation process:

$$i(t) = A_1 e^{\left(-\frac{t}{\tau_1}\right)} + A_2 e^{\left(-\frac{t}{\tau_2}\right)} + A_3 e^{\left(-\frac{t}{\tau_3}\right)} + \cdots \quad [12]$$

This is a textbook representation of a multi-exponential decay, where A_k are amplitude coefficients and τ_k are characteristic time constants. Processes such as double-layer charging, specific ion adsorption, or diffusion-driven relaxations can each contribute with different τ_k and varying weights(1, 2). Extracting the underlying amplitudes and time constants from $\mathrm{i(t)}$, is classically ill-posed, the Laplace transform of a sum of exponentials is a rational function, and inverting it is numerically unstable and highly sensitive to noise (19). Ambiguities in separation arise especially

when τ_k values are close (spaced within a factor <2–3, *vide infra*), amplitudes differ greatly, or weak components fall below the noise threshold. Complementary observables can mitigate this ambiguity. Throughout this work, we use 'complementary' to describe observables with distinct or weakly overlapping sensitivity profiles, recognizing that strict orthogonality or independence, in the mathematical sense, is neither required nor realistically attainable in most experimental systems. For instance, pairing current measurements with time-resolved attenuated total reflectance-surface - enhanced infrared absorption spectroscopy (ATR-SEIRAS) at a specific wavenumber yields two projections of the same underlying kinetics onto distinct measurement spaces. These complementary modalities, while not necessarily mathematically orthogonal, differ in their sensitivity profiles and effectively expand the rank of the measurement matrix (20–22). When applied together, they transform the inverse problem from a scalar inversion into a geometrically richer projection across observable dimensions; particularly in high-dimensional spectroelectrochemistry, where time-resolved spectra (i.e., multiple wavelengths measured at the same time) track dynamic signatures across wavelengths.

1.4 Separability from Classical Signal Processing Literature

From classical signal processing, we know that separability relies on the presence of contrast in (i) time, (ii) spectrum (wavenumber), (iii) amplitude, or (iv) response shape, which are typically system-inherent but can nevertheless be accessed or manipulated by probe selection (spectroscopic technique) and design (e.g., perturbation waveform input). Even when response functions are formally linearly independent, overlapping bandwidths in the response frequencies, similar decay constants τ, or shared observability channels, can render practical separation ambiguous or unstable (23). Although separability in experimental systems is often inherently approximate and qualitative, establishing a (semi-)quantitative framework remains valuable for guiding experimental design and data interpretation. In the idealized case when processes are uncoupled and follow first-order kinetics, the total response may be expressed as the sum of exponentials, each with a distinct time constant and weight as in Eq. 4. The corresponding frequency-domain representation of each first-order process is:

$$H(\mathrm{t}) = \frac{1}{1+(2\pi f \tau)^2} \quad [13]$$

with a cutoff frequency

$$f_{cutoff} = \frac{1}{2\pi\tau} \quad [14]$$

Fast processes (small τ**)** have a high f_{cutoff}, yielding broader frequency responses, which respond (also) to the high frequency square-wave harmonics (4, 23). Slow processes (large τ**)** on the other hand, have a narrower frequency response, and only respond to low-frequency content, effectively acting as low-pass filters. When values differ by a factor of 5 or more, their frequency bands become quasi-orthogonal, ensuring minimal interference, and temporal separation is straightforward. This is a widely used (heuristic) rule of thumb, stemming from signal processing linked to the difference in the systems' -3 dB bandwidths (i.e., where the response amplitude drops to $\frac{1}{\sqrt{2}}$ of its maximum) (23). When the ratio drops to $\frac{\tau_2}{\tau_1} \sim 3$, partial mixing occurs, where components can in theory still be distinguished by dimensionality reduction techniques, but the separation is less clean. Below a ratio of ~2, the separation problem becomes ill-posed, and separability becomes ambiguous with mode blending increasing significantly. As the ratio narrows toward unity, components blend, and the inverse problem becomes numerically unstable. It must be noted that these rules of thumb are heuristic, and only holds when all processes (i) contribute measurably to the observable, (ii) their amplitudes are sufficiently large (e.g., not buried in noise), (iii) the processes are uncoupled, and (iv) the measurement is either noise-free or with sufficiently high signal-to-noise ratio. It must also be noted that important nuances to these rules exist in the context of our framework, as will be discussed in the following Section (Section 2).

2. Retrievability and Disentanglement of Coupling

Having established how the input waveform defines which dynamic processes are excited through its frequency content, amplitude, and absolute potential, and how complementary observables expand the rank of the measurement space to enable richer recovery of latent dynamics, we now address the foundational question of separability; under what conditions can overlapping interfacial responses be disentangled in DRS?

2.1 Simulation Framework

To quantify separability in dynamically perturbed latent systems, we create a framework that allows us to systematically analyze the visibility or resolvability of spectral-dynamic systems under a wide range of contributing variables and data analysis techniques.

We define a dynamic system consisting of *n* electrochemically active components. Each component *i* evolves a time-dependent response function Ri(t), driven by an external modulated signal S(t), and is linearly or nonlinearly coupled to other components in the system. The governing equation is defined below, where τ_i is the characteristic time constant of component *i*, e.g., related to a kinetic process (adsorption, double-layer charging, etc.), A_i is the amplitude scaling factor of the electrochemical response, for example due to concentration effects, α_{ij}, and β_{ij} are the linear, and nonlinear coupling strength from component j to i, respectively, and N_{ij} is the nonlinear interaction function defined per pair (i, j). Diagonal terms of α_{ii}, and β_{ii} are always zero and both matrices are symmetric (e.g., $\alpha_{ij} = \alpha_{ji}$).

$$\frac{d\hat{R}_i}{dt} = \frac{1}{\tau_i}\left(S(t) - \mathrm{f}_i(\hat{R}_i(t))\right) + \sum_{j\neq i}\alpha_{ij}\left(R_j(t) - \hat{R}_i(t)\right) + \sum_{j\neq i}\beta_{ij}\,\mathrm{N}_{ij}\left(R_j(t), \hat{R}_i(t)\right) \quad [15]$$

Where the final $\mathrm{R_i(t)} = \mathrm{A_i} \cdot \hat{R}_i(\mathrm{t})$ so that the internal timescale and dynamics are controlled only by τ_i, f_i, and coupling, and the amplitude A_i only affects the final observable Ri(t). Term f_i is a vector of functions to represent nonlinear excitation (response), for example when systems saturate, rectify, or otherwise filter their responses. Here, we consider only saturation ($\mathrm{f_i} = \tanh(R_i - R_j)$) or nothing ($\mathrm{f}_i(\hat{R}_i(t)) = \hat{R}_i(t)$), in which case the response function is linear. The linear coupling term is written as $R_j(t) - R_i(t)$ to represent diffusive-like or relaxation-based coupling, which represents drift toward equilibrium. If $R_j(t) > R_i(t)$, the term is positive: component i is pulled up toward j, and vice versa. In either case, the system tends to reduce the mismatch between i and j, which is important when modeling coupled dynamic systems aiming to synchronize or equilibrate through common mass/charge balances or influencing each other via potential differences or gradients which is the situation we aim to model. We define three core nonlinear interaction modes $\mathrm{N_{ij}}$: sigmoidal threshold: $\mathrm{N_{ij}} = \frac{1}{1+e^{-k(x-\theta)}}$; $\mathrm{N_{ij}} = \tanh(R_i - R_j)$, and stochastic spiking $\mathrm{N_{ij}}(R_j, R_i) = \epsilon \cdot \delta(\mathrm{t} - t_k)$, where $t_k$is a random time point and ϵ is a burst amplitude which introduces rare, sharp coupling events. Each $\mathrm{N_{ij}}$ may be chosen independently, enabling heterogeneous nonlinearities within the same simulation. The multiplicative case is positive feedback, which can create runaway growth or state-dependent amplification, if coupled with saturation or thresholds elsewhere, can produce switching behavior. The saturating case introduces bounded nonlinearity, which yields smooth thresholds and S-shaped response curves, mimicking sigmoid kinetics, which in dynamical systems are classic precursors to bifurcations. The stochastic nonlinearity introduces noise-driven jumps, metastable states, and event-triggered transitions. Even in a deterministically monostable system, adding a stochastic kick can simulate bifurcation-like state transitions. The coupling matrices α and β are symmetric by assumption (i.e., $\alpha_{ij} = \alpha_{ji}$, $\beta_{ij} = \beta_{ji}$) which simplifies computation, though we acknowledge that realistic systems can break symmetry. Even with linear alpha matrices, if β ≠ 0, the nonlinear terms dominate behavior once responses grow. This thus gives us a basic framework to explore emergent nonlinear dynamics by scanning beta strength or nonlinear form. We explicitly simulated three activation regimes by using binary modulation masks

applied to the system's input functions: off/off (no components receive input), off/on (only one component is modulated), and on/on (multiple components are modulated simultaneously). That is, for n = 3, binary multipliers [0, 0, 0], [0, 1, 0], and [1, 1, 1] or [1, 0, 1] are applied to simulate the off/off, off/on, and on/on regimes. Each mask is applied element-wise during the generation of component-specific response functions, effectively controlling which components are "active" in response to S(t), allowing to probe recoverability under different coupling and observability scenarios.

Each component i has a unique or shared spectral fingerprint $F_i(\nu)$ (see Figure S1), such that the total system spectrum over time is given by

$$D(v, t) = \sum_{i=1}^{n} F_i(\nu) \cdot R_i(\mathrm{t}) \quad [16]$$

Or in matrix form:

$$D[\nu \times \mathrm{t}] = F[\nu \times n] \times R[n \times t] \quad [17]$$

Where ν is energies, n is number of components, and t is times.

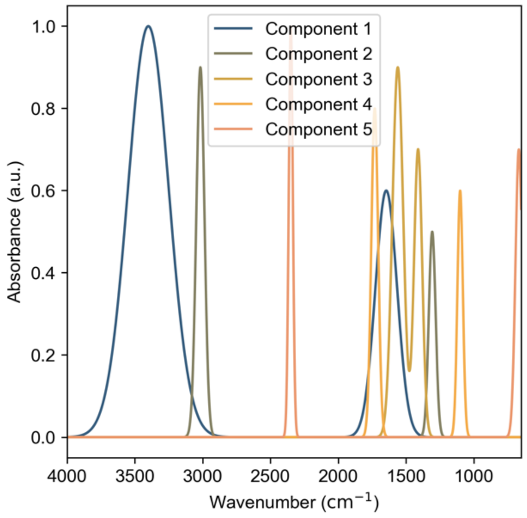

Figure S1. The selected spectral components (where fully overlapping components are tested as 3x liquid water (Component 1), partially overlapping components are tested as liquid water, methane gas (Component 2) and carboxylate (Component 3), and non-overlapping components as liquid water, formyl (Component 4), and CO_2 gas (Component 5)).

2.2 Disentangling Responses

To evaluate the capability of different analytical strategies in resolving overlapping, dynamic spectroelectrochemical signals, we use synthetic data generated by the governing equation Eq. S15. Each simulated dataset encodes multiple electrochemical components with known time constants τ_i, scaled amplitudes A_i, and internal states $\hat{R}_i(t)$. Our objective is to recover these latent variables; particularly τ_i, and R_i - as well as the spectral fingerprints $F_i(\nu)$, and ultimately the number of distinguishable components.

We assess three families of analysis tools:

1. Phase-sensitive detection (PSD) for frequency-domain demodulation,
2. Principal component analysis (PCA) for unsupervised dimensionality reduction, and
3. Autoencoders (AE) for nonlinear manifold learning.

A simple test case is demonstrated below for each with a square wave modulation of a period of 50 s, 3 components (Components 1-3 from Figure S1) with τ values of 1, 5, 10, a linear response, and no coupling.

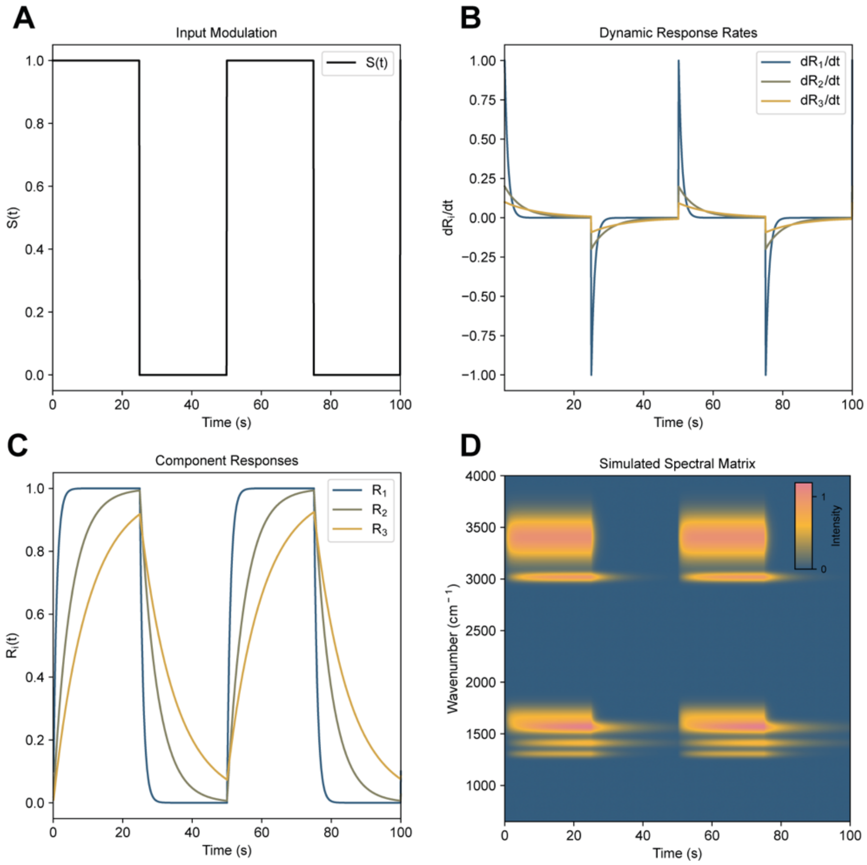

Figure S2. Overview of the A) input signal S(t), B) the solved response rates d$\hat{R}_i$/dt, and C) resulting component response internal states $\hat{R}_i$, and D) the resulting spectral matrix for a square wave input. The system includes three components (R_1–R_3) with τ = 1, 5, 10 s and equal amplitudes (i.e., $\hat{R}_i$ = R_i).

2.2.1 Phase Sensitive Detection

Modulated excitation–phase sensitive detection (ME-PSD) is a classical approach designed to improve signal-to-noise ratio in LTI systems(24). It assumes that the system is probed with a periodic sinusoidal input angular frequency ω:

$$E(t) = E_0 \sin(\omega t) \quad [18]$$

yields a system response,

$$R(t) = A \sin(\omega t + \phi) \quad [19]$$

where amplitude A and phase shift ϕ are extracted via quadrature demodulation:

$$A = \frac{2}{T}\int_0^T R(t)\sin(\omega t)\,dt\,, \qquad B = \frac{2}{T}\int_0^T R(t)\cos(\omega t)\,dt \quad [20]$$

$$|R(\omega)| = \sqrt{A^2 + B^2}, \qquad \phi(\omega) = \arctan\ 2(B, A) \quad [21]$$

The response is separated into in-phase (I) and quadrature (Q) components, forming a complex-valued spectral matrix:

$$\widetilde{D}[\nu \times t] = I[\nu] + iQ[\nu] \quad [22]$$

From these, the amplitude spectrum is:

$$A(\nu) = \sqrt{I(\nu)^2 + Q(\nu)^2} \quad [23]$$

And the phase spectrum is:

$$\phi(\nu) = \arctan\left(\frac{Q(\nu)}{I(\nu)}\right) \quad [24]$$

This decomposition allows identification of which spectral features are most responsive to the applied modulation and their associated phase lag. However, PSD cannot distinguish whether a given phase delay arises from kinetic lag or from a reduced amplitude response at that frequency. Furthermore, because PSD isolates components at a specific reference frequency, it inherently filters out aperiodic or broadband behaviors, obscuring nonstationary or emergent dynamics.

PSD was employed using a custom NumPy-based (version 2.1.3), with sinusoidal references generated at the fundamental modulation frequency. The modulation period was set to match the input modulation period, and the number of phase bins used for reconstruction was 12. Spectral intensities were decomposed into real (cosine) and imaginary (sine) components by projecting each time trace onto reference sine and cosine functions. From these, we calculated the amplitude spectrum, phase spectrum, and stacked phase-resolved spectra, enabling the identification of modulated vibrational features and their phase relationships relative to the input waveform.

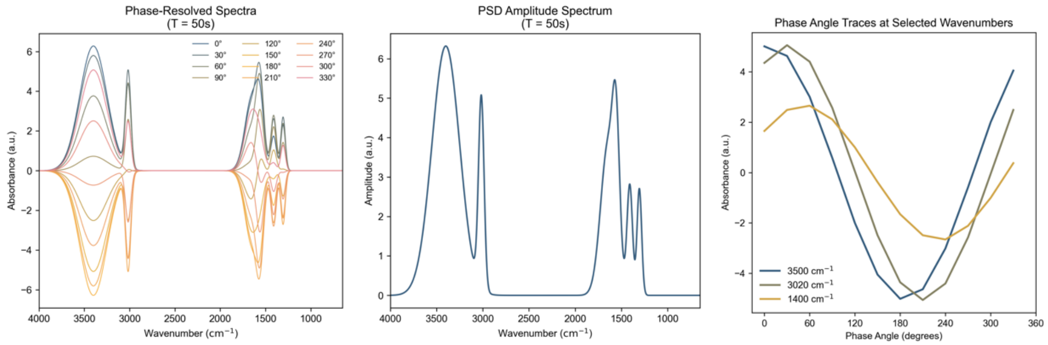

Figure S3. PSD decomposition of the spectral matrix in Figure S2; (**A**) Phase-resolved spectra and (**B**) amplitude spectrum of the spectral matrix presented in Figure S2D. (**C**) Traces of the phase angle spectra at wavenumbers 3500, 3020, and 1400 cm^{-1}, characterizing Components 1, 2, and 3, respectively.

Especially under conditions where response functions are nonlinear, nonstationary, or coupled, classical methods fall short. ME-PSD is a key example that shows the power of combining spectroscopy with pulsed modulation, but in this approach, period averaging is considered a key step to increase signal-to-noise ratio (25–27). While theoretically rigorous for true, ideal LTI systems, this approach averages out transients, filters out non-periodic structure, and cannot distinguish kinetic, from amplitude origins. These limitations that are intrinsic to the method's architecture and not easily overcome within its paradigm. We note that while our implementation and discussion here focus on first-harmonic detection, higher harmonics or continuous wavelet decomposition could be used to potentially access more complex nonlinear behavior (28).

2.2.2 Principal Component Analysis

PCA is perhaps one of the most often employed dimensionality reduction approaches, but even for this approach, we must be aware of our assumptions and limitations if we wish to analyze and understand coupled dynamics. PCA provides a model-free method to decompose the spectral data matrix $D[\nu \times \mathrm{t}]$ into orthogonal temporal and spectral components via singular value decomposition:

$$D = T \cdot P^{T}, \qquad [25]$$

Here, T contains the time-dependent score vectors (temporal profiles of each component) and P contains the loading vectors (the spectral patterns associated with each component). The principal components are orthogonal by construction, meaning:

$$\langle PC_i, PC_j \rangle = 0 \; for \; i \neq j \qquad [26]$$

This orthogonality is statistical, not physical, in the sense that it is based on the covariance matrix of the data. It ensures that each component explains a unique axis of variance in the dataset, but it does not guarantee mechanistic independence. Indeed, two physically coupled processes may be represented in PCA as distinct components if their responses are uncorrelated in time as will be shown later on, and two independent processes may appear as mixed components if their time dependencies partially overlap, their amplitudes are similar, or there is spectral overlap. An example of this can be seen in the PCA decomposition of the spectral matrix presented in Figure S2D, which is shown in Figure S4. We consider Components 1, 2, and 3 which have partial spectral overlap, the same amplitudes, and which (while having quite distinct τ values) share a similar time profile evolving over the same period in time. We can see that neither component is fully recovered in the scores or loadings.

PCA can cleanly distinguish for even the latter case in the time domain, provided their kinetic signatures remain separable. The interpretive strength of PCA thus hinges on the temporal structure of variance. In ideal scenarios, for uncoupled nonlinear responses with non-overlapping timescales, PCA will identify distinct physical processes as orthogonal modes. However, as coupling increases, or when signal-to-noise becomes limiting, components no longer align neatly with isolated mechanisms. Instead, they may blend into shared axes or require multiple principal components to represent what is fundamentally one emergent behavior. For such cases, PCA becomes a qualitative diagnostic rather than a mechanistic dissection. Cross-validation, for example, with other data analysis approaches such as perturbation shape variation or amplitude-dependent probing, can help to mitigate these ambiguities, but interpretation always requires mechanistic anchoring.

We applied PCA using sklearn.decomposition.PCA from the scikit-learn 1.6.1 library, with the number of components set to match the number of components.

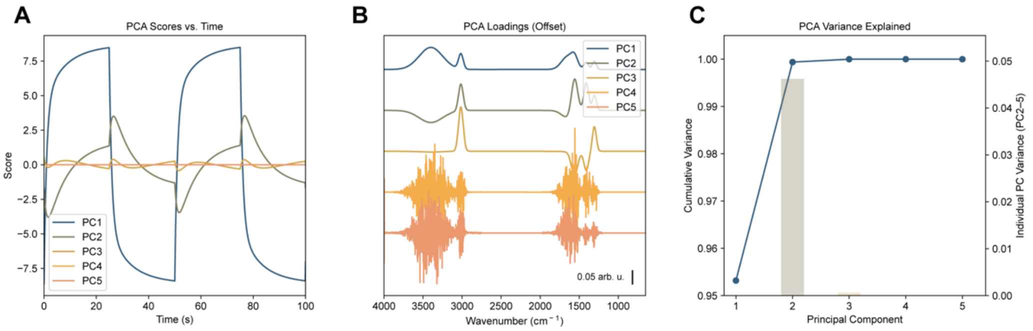

Figure S4. PCA decomposition of the spectral matrix in Figure S2; (**A**) PCA scores over time for the first five principal components, (**B**) PCA loadings plotted with vertical offset, showing spectral features associated with each component, (**C**) Cumulative and individual variance explained by each PCA mode.

2.2.3 Autoencoder

Autoencoders are unsupervised neural network architectures designed to learn compact, nonlinear representations $\mathcal{Z}^{(k\times t)}$ of input data $\mathcal{D}^{(v\times t)}$ (29, 30) where v denotes the number of input features (e.g., wavelengths in our spectra), t denotes the number of temporal data points, and k < v indicates dimensionality reduction. An AE consists of two main parts, the encoder:

$$f_\theta: \mathcal{D}^{(v\times t)} \rightarrow \mathcal{Z}^{(k\times t)}, \qquad [27]$$

which maps high-dimensional input data into a lower-dimensional latent space $\mathcal{Z}$ through a set of mathematical transformations f_θ, where θ denotes a set of weight and biases, and the decoder:

$$g_\phi: \mathcal{Z}^{(k\times t)} \rightarrow \widehat{\mathcal{D}}^{(v\times t)} \qquad [28]$$

which reconstructs the input from the latent variables back into observation space through a function g_ϕ, where ϕ represents the decoder parameters that perform the inverse transformation of the encoder. The network is trained to minimize the reconstruction loss $\mathcal{L}(\theta, \phi)$:

$$\mathcal{L}(\theta, \phi) = \frac{1}{N} \| \mathcal{D}^{(v\times t)} - \widehat{\mathcal{D}}^{(v\times t)} \|_2^2 \qquad [29]$$

Unlike PCA, which enforces linear orthogonality onto non-necessarily physically linear systems, AEs can learn nonlinear manifolds and accurately uncover hidden structure even in highly coupled or nonlinear systems using activation functions layers, which apply nonlinear transformations within f_θ and g_ϕ. In this way, the latent dimensions $\mathcal{Z}_i$ can reflect emergent behaviors, encode separability across time or spectral patterns, and enable unsupervised clustering of system dynamics.

In the context of DRS, autoencoders may therefore offer a promising pathway to extract distinct dynamic modes that are not linearly separable, by disentangling processes with overlapping and/or coupling spectral signatures in the latent space. For example, coupled processes with overlapping spectra and nonlinear interactions may be encoded in separate latent vectors if they manifest distinct temporal structure. By examining trajectories in latent space, discontinuities, transitions, or nonlinear phase evolution can be visualized. However, several methodological considerations must be addressed when applying autoencoders-based dimensionality reduction to spectroscopic data analysis. The interpretability of the latent representations is not inherently guaranteed, as the nonlinear transformations employed during compression may obscure direct connections between latent dimensions and underlying physical processes. The compressed representation, while mathematically optimal for reconstruction, may not necessarily correspond to physically meaningful variables or processes. Advanced methodological approaches, such as physics-informed autoencoders (31), which incorporate domain-specific penalty terms in the loss function to constrain latent representations toward physically plausible behaviors, may be necessary to ensure meaningful decomposition. Additionally, the selection of latent dimensionality presents a non-trivial optimization challenge. While the present analysis shown below constrains the number of latent dimensions to match the number of components in our theoretical framework, real experimental systems may require more sophisticated dimensionality selection strategies. For instance, while complex electrochemical systems exhibit hierarchical dynamics across multiple timescales, excessive latent dimensionality may lead to overfitting and incorporation of noise-driven artifacts rather than genuine physical features. Systematic approaches for optimal latent dimension selection, potentially incorporating information-theoretic criteria or cross-validation strategies, represent important areas for future methodological development.

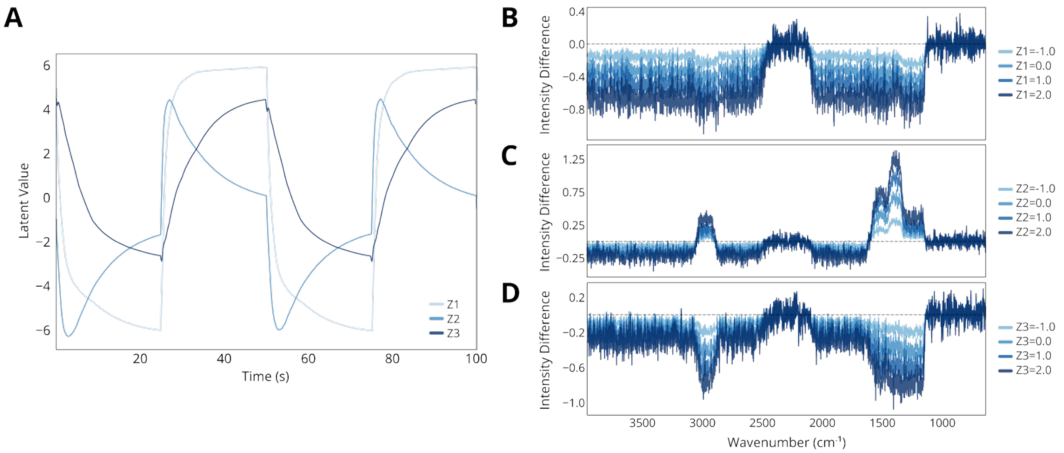

Figure S5. (**A**) Latent dimensions $\mathcal{Z}_i(t)$ from AE decomposition of the spectral matrix presented in Figure S2, and the reconstructed spectral variations obtained by traversing each latent dimension $\mathcal{Z}_i$ across values of -1, 0, 1, and 2, with difference spectra calculated relative to $\mathcal{Z}_i$ = -2 for (**B**) i = 1, (**C**) i =2, and (**D**) i = 3

In this work, we employ a basic feedforward autoencoder implemented in PyTorch 2.7.0 to explore latent representations of time-resolved spectral data. The model consists of a two-layer encoder and decoder with ReLU activations, trained to minimize mean squared reconstruction error. The latent dimensionality was set to match the number of components to allow comparison with PCA-based representations. The implementation is intentionally minimal, intended to illustrate fundamental capabilities and limitations rather than to exhaust the full potential of deep learning architectures. While more advanced architectures such as variational autoencoders (VAEs)(32), physics-Informed autoencoders (PIAEs)(33), or hybrid deep-learning models that combine recurrent neural networks (RNNs) (34)-based architectures with autoencoders could offer superior performance in capturing nonlinear, time-evolving system dynamics, these approaches often involve more complex training regimes, hyperparameter tuning, and stability considerations. A full exploration of these methods is beyond the scope of the present study, but we highlight their relevance for future work aiming to disentangle rich, coupled behaviors in electrochemical systems. The code employed, available in the Vogt lab´s GitHub (35) reflects the structure and training used for the results presented in the main text. The evolution of the loss function across multiple epochs is shown in Figure S6.

Figure S5A presents the temporal evolution of the latent dimensions extracted from autoencoder compression of the spectral dataset introduced in Figure S2. The latent feature trajectories exhibit single exponential decay characteristics that closely mirror the kinetic profiles presented in Figure S2C, accurately reproducing the temporal response to the simulated external perturbation. This contrasts markedly with the results retrieved from PCA, where the scores associated to PC3 (Figure S4A) displays polynomial-like temporal evolution that does not correlate with the exponential kinetics of the simulated system, indicating potential artifacts introduced by the orthogonality constrain.

To elucidate the spectral contributions associated with each latent dimension trajectory, we employed latent space traversal analysis (32), providing interpretive information analogous to PCA loadings. This approach systematically varies individual latent dimensions $\mathcal{Z}_i$ while maintaining others constant, enabling direct visualization of the spectral changes encoded by each latent variable. Figures S5C-D present the reconstructed spectral variations obtained by traversing each latent dimension $\mathcal{Z}_i$ across values of -1, 0, 1, and 2, with difference spectra calculated relative to $\mathcal{Z}_i$ = -2. This traversal analysis reveals the specific spectral features and wavelength regions that each latent dimension encodes, facilitating physical interpretation of the compressed representation and enabling comparison with the corresponding eigenspectra from PCA. We note that a quick qualitative comparison between Figure S4B and Figure S5B-D, i.e., PCA eigenspectra vs AE

traversed reconstructed spectral variations shows that PCA retrieves far more realistic components.

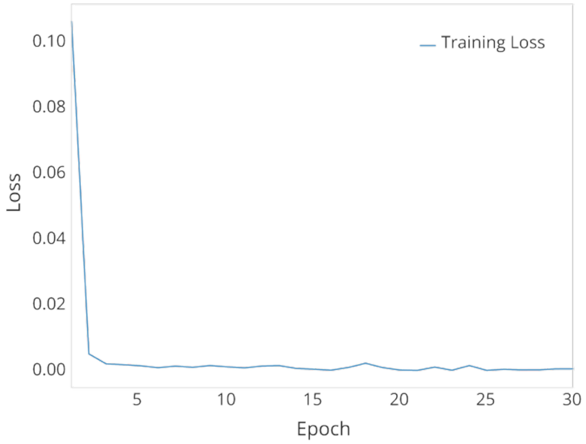

Figure S6. Training convergence of the autoencoder reconstruction loss. The mean squared error between original and reconstructed spectra decreases rapidly during the initial training epochs and reaches a stable minimum, indicating successful learning of the spectral manifold structure.

In the next section, we further evaluate the effectiveness of these techniques in recovering ground-truth kinetic parameters and latent dynamics across a range of simulated systems, varying noise, amplitude, and relaxation time scales. We will focus on PCA for the reasons described above, but also extensively compare AE and PCA for coupled versus noncoupled systems.

2.3 Separability Criteria

To obtain information about our system, following the governing Eq. 15, we wish to re-obtain the different variables from the equation: characteristic time constants τ_i, the response functions R_i, amplitudes A_i, spectral fingerprints of each component $F_i(\nu)$, or the number of components i. We will focus on τ_i, $R_i(t)$ as they are the primary descriptors of individual component dynamics, governing both the characteristic response speed and the evolution of the system over time. Our goal was to evaluate how well these variables embedded in the synthetic datasets can be inferred from signal decompositions.

2.3.1 Linear and Uncoupled Systems

We assess retrievability of τ_i from latent trajectories obtained by PCA (principal component loadings T_k(t)), AE (latent dimensions $\mathcal{Z}_i$(t)), and PSD (frequency-resolved harmonics). In the absence of coupling and nonlinearity, the governing equation Eq. 15 simplifies to:

$$\frac{d\hat{R}_i}{dt} = \frac{1}{\tau_i}\left(S(t) - \hat{R}_i(t)\right) \qquad [30]$$

For each time-resolved latent trace (i.e., for PCA or AE), we exploit the analytical solution of this first-order ordinary differential equation under modulated input S(t) to estimate τ_i by fitting through scipy.optimize.curve_fit (1.15.2) to the exponential rise function:

$$y(t) = A(1 - e^{-kt}) + C \qquad [31]$$

A is the amplitude of the response (capturing the dynamic magnitude), k is the rate constant, such that τ=1/k, C is a vertical offset, absorbing baseline signal levels or drift. While A and C are fit parameters, they are not used in our evaluation of kinetic fidelity. The focus is solely on recovering the correct timescale structure, not absolute signal intensity or baseline values. All fits were

smoothed using a Gaussian filter (σ = 1) to reduce noise sensitivity. The fitted time constant for each PC is then defined as τ_i = 1/k, and fits were only accepted when convergence was achieved with k>0 and residuals were within 5% of the signal amplitude. The modulation onset t_0 was identified from the input waveform S(t) (e.g., first transition for square/sawtooth, local minimum for sine), and the fitting window was defined as [t_0, t_0 + Δt], with Δt = 5 s. Fits were accepted when k>0 and residuals were <5% of amplitude. To account for arbitrary scaling in latent spaces, τ values were normalized relative to the slowest component:

$$\vec{r}_{true} = \frac{\vec{\tau}_{true}}{\tau_{true,1}},\ \vec{r}_{fit} = \frac{\vec{\tau}_{fit}}{\tau_{fit,1}},\ \varepsilon_{\tau} = \left\|\vec{r}_{true} - \overline{\vec{r}_{fit}}\right\|_2 = \sqrt{\sum_{i=1}^{j}(\vec{r}_{true,i} - \vec{r}_{fit,i})^2} \quad [32]$$

This yields a dimensionless error quantifying the fidelity of relative timescale recovery. A perfect match yields ε_τ=0. Higher values indicate distortions in the inferred dynamic ordering or separation between components.

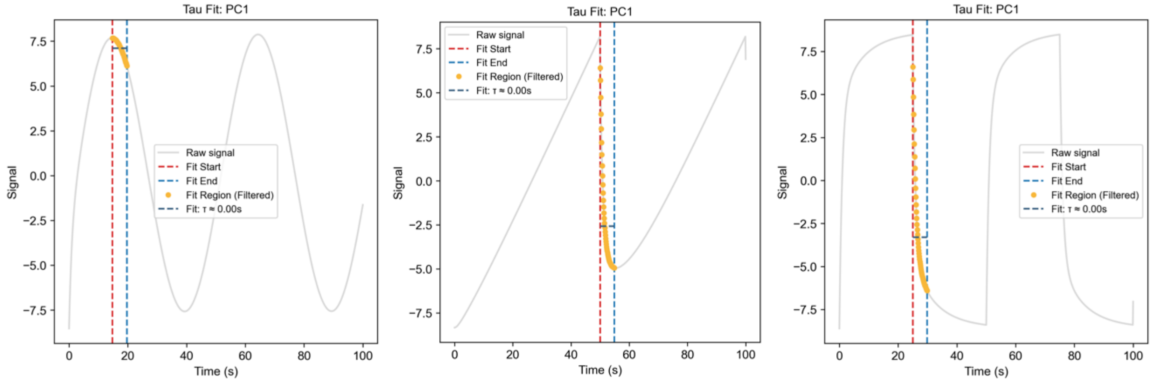

Figure S7. Examples of the fit τ_i values for the sine, sawtooth, and square wave simulations presented in Figures S8-10.

For PSD, τ_i was retrieved analytically from the phase lag via:

$$\tau_i = \frac{\tan(\phi_i)}{\omega} \quad [33]$$

2.3.2 Nonlinear and Coupled Systems

For coupled or nonlinear systems, responses include contributions from direct dynamics and interactions across components. Here, τ_i no longer maps cleanly to a single frequency or first-order kernel, and PSD becomes ill-defined: multiple frequencies and nonlinear responses mix into each observable. Therefore, PSD is not considered in this regime. Instead, we quantify retrievability via projection of the true system responses and governing equation terms onto the latent basis (from PCA or AE):

$$R_i(t) = \sum_k b_k \cdot Z_k(t) \quad [34]$$

We compute the R^2 coefficient for each projection, comparing latent reconstruction of:

- the full system responses $R_i(t)$,
- linear coupling terms $\sum_{j\neq i} \alpha_{ij}\left(R_j(t) - R_i(t)\right)$,
- the nonlinear interactions $\sum_{j\neq i} \beta_{ij}\, \mathrm{N}_{ij}\left(R_j(t), R_i(t)\right)$,
- and the τ-scaled driving term $\frac{1}{\tau_i}\left(S(t) - \hat{R}_i(t)\right)$

This allows a direct comparison of PCA and AE in terms of their ability to disentangle entangled dynamics. To summarize retrievability across components, we compute the root mean square of the best R^2 values between each true Ri and its best-matching latent trajectory:

$$R_{i\,retrieval} = \sqrt{\frac{1}{n}\sum_{i=1}^{n}(R_{best}^{2}(R_i \to PC_j/Z_j))^2} \qquad [35]$$

2.4 Test Cases

2.4.1 Uncoupled Linear Systems

To explore the influence of modulation waveform and kinetic diversity on system observability, we simulated uncoupled systems with three components (τ = 1, 5, 10 s; amplitudes = 1) under three canonical input shapes: sine, sawtooth, and square waves. These synthetic test cases provide a clean benchmark for retrievability across decomposition methods. For each case, we generated time-resolved spectral matrices D[v,t], derived from the dynamic responses Ri(t), and applied PCA, PSD and AE to assess how accurately the true timescales and response functions could be recovered, providing a clean benchmark for evaluating how well different analysis methods capture intrinsic dynamics.

Figures S8–S10 show the results for sine, sawtooth, and square modulations, respectively. Each figure presents the input modulation function S(t), solved component responses Ri(t), PCA scores, loadings, and variance explained, PSD phase angle spectra and phase lag, and AE latent dimensions $\mathcal{Z}$.

The corresponding τ ratio errors (ε_τ) and $R_{i\,retrieval\,scores}$ are summarized in Table S1. Comparing the different waveforms, square wave modulation yielded the lowest τ-ratio errors (PCA: 107, AE: 341), indicating superior recoverability of characteristic time constants. This is attributed to the abrupt transitions in S(t), which generate temporally sharp response differences, enabling PCA to isolate orthogonal modes associated with relaxation kinetics. However, square waves slightly compromise R_i reconstruction fidelity (PCA score = 0.81), likely due to their abruptness inducing non-smooth latent encoding. Sawtooth input, by contrast, provided the highest retrieval scores for $R_i(t)$ (AE: 0.98, PCA: 0.90), as its continuous ramping enables the latent space to track temporal evolution smoothly. This comes at the cost of slightly higher τ-ratio errors (PCA: 147, AE: 809), reflecting a tradeoff between accurate kinetic separation and physical dynamic fidelity. Sine input performed worst across all metrics (τ-ratio error: PCA = 3802, AE = 634), despite respectable R_i retrieval (AE: 0.95), highlighting its vulnerability to phase overlap and harmonic entanglement. Smooth modulation blurs kinetic distinctions, especially when timescales partially overlap, hampering both separation and τ extraction.

Table S1. Overview of the calculated τ ratio error (ε_τ) (for PSD, PCA, and AE), and the $R_{i\,retrieval\,score}$(for PCA and AE) from Figures S8-10 (i.e., with 3 components, τ = 1, 5, 10 s; amplitudes = 1).

	τ ratio error (ε_τ)			$R_{i\,retrieval\,score}$	
Input function	PSD	PCA	AE	PCA	AE
Sine	9.84	3802	634	0.83	0.95
Sawtooth	9.84	147	809	0.90	0.98
Square	9.84	107	341	0.81	0.97

This highlights a fundamental distinction: maximizing τ-rank separability (i.e., variance-aligned decomposition) and reconstructing true dynamics $R_i(t)$ are non-coincident objectives. Square modulation sharpens τ resolution but can compress latent trajectories; sawtooth favors smoother and more physical encoding of system evolution. Furthermore, despite the system being fully linear and uncoupled, PSD analysis failed uniformly across waveforms, returning the same τ error regardless of modulation (ε_τ = 9.84). This arises because PSD assumes a one-to-one

correspondence between input modulation and output phase lag (i.e., τ_i=tan (ϕ_i)/ω, which does not hold in practice when multiple overlapping responses contribute to the signal. Even in uncoupled systems, superposition of dynamic signatures distorts phase interpretation. This limitation is exacerbated in nonlinear or coupled regimes, where response mixing and harmonic generation further undermine phase-based inference. As such, PSD is excluded from further retrievability evaluation.

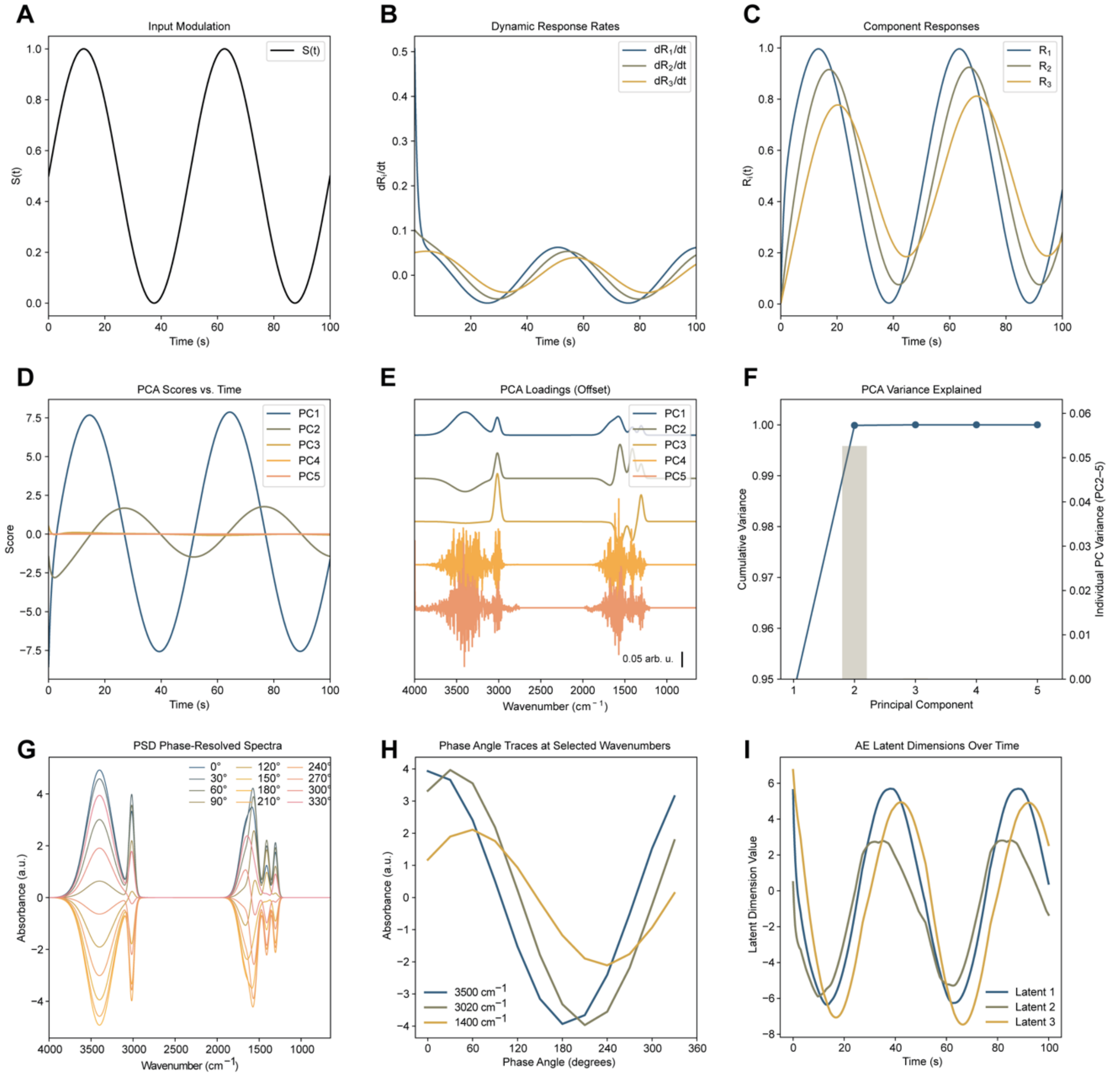

Figure S8. Overview of the input signal, component responses, and principal component analysis (PCA) of the resulting spectral matrix for a sine-wave input. The system includes three components (R_1–R_3) with τ = 1, 5, 10 s and equal amplitudes. (**A**) Input modulation function S(t). (**B**) Solved time-derivatives of the response functions dR_i/dt. (**C**) Response functions $R_i(t)$. Decomposition by PCA, PSD, and AE; (**D**) PCA scores over time for the first five principal components, (**E**) PCA loadings plotted with vertical offset, showing spectral features associated with each component, (**F**) Cumulative and individual variance explained by each PCA mode. (**G**) Phase resolved spectra from PSD, (**H**) Traces of the phase angle spectra at wavenumbers 3500, 3020, and 1400 cm^{-1}, characterizing Components 1, 2, and 3, respectively, and (**I**) latent dimensions 1-3 from AE.

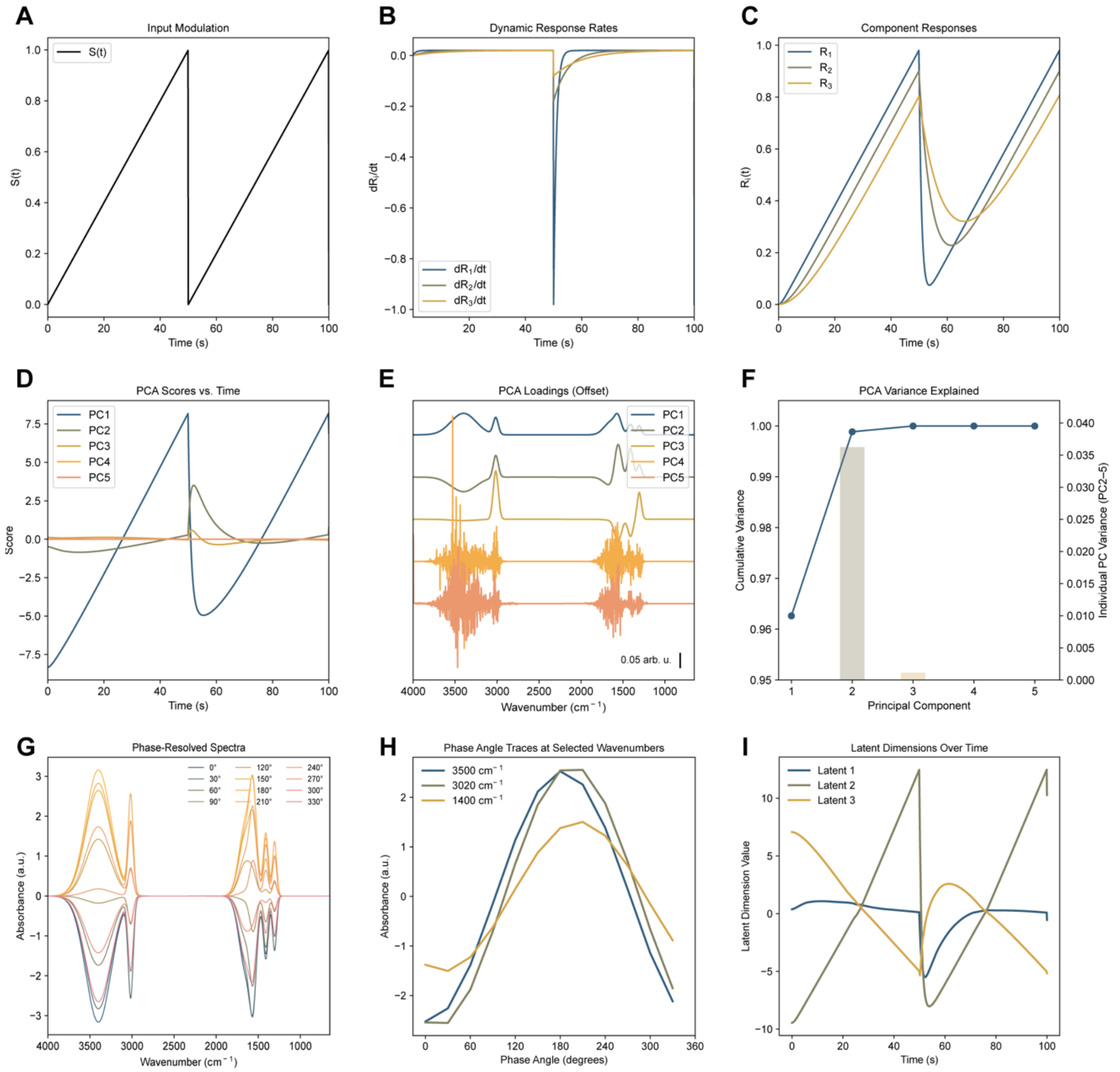

Figure S9. Overview of the input signal, component responses, and principal component analysis (PCA) of the resulting spectral matrix for a sawtooth-wave input. The system includes three components (R_1–R_3) with τ = 1, 5, 10 s and equal amplitudes. (**A**) Input modulation function S(t). (**B**) Solved time-derivatives of the response functions dR_i/dt. (**C**) Response functions $R_i(t)$. Decomposition by PCA, PSD, and AE; (**D**) PCA scores over time for the first five principal components, (**E**) PCA loadings plotted with vertical offset, showing spectral features associated with each component, (**F**) Cumulative and individual variance explained by each PCA mode. (**G**) Phase resolved spectra from PSD, (**H**) Traces of the phase angle spectra at wavenumbers 3500, 3020, and 1400 cm^{-1}, characterizing Components 1, 2, and 3, respectively, and (**I**) latent dimensions 1-3 from AE.

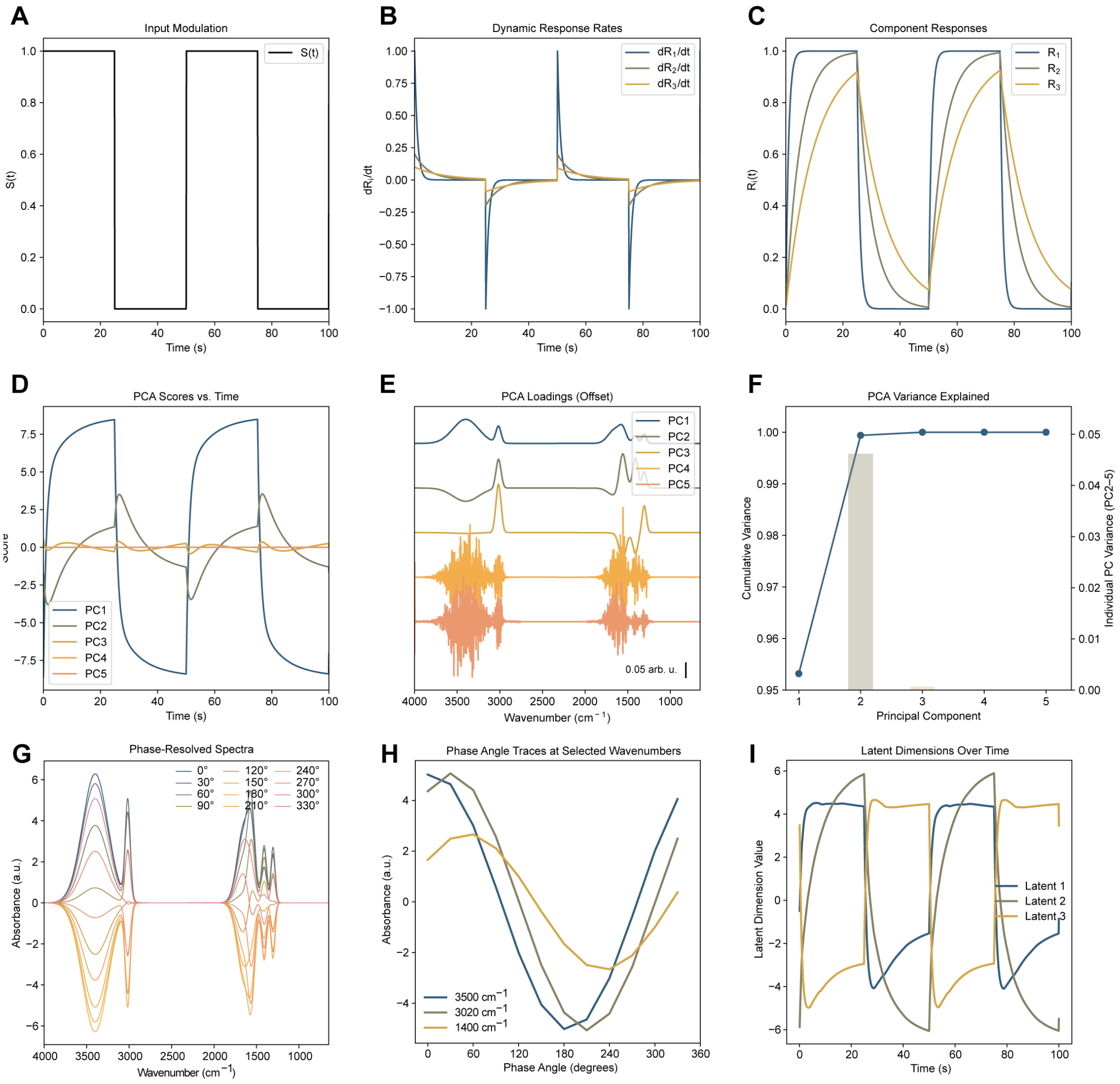

Figure S10. Overview of the input signal, component responses, and principal component analysis (PCA) of the resulting spectral matrix for a square-wave input. The system includes three components (R_1–R_3) with τ = 1, 5, 10 s and equal amplitudes. (**A**) Input modulation function S(t). (**B**) Solved time-derivatives of the response functions dR_i/dt. (**C**) Response functions $R_i(t)$. Decomposition by PCA, PSD, and AE; (**D**) PCA scores over time for the first five principal components, (**E**) PCA loadings plotted with vertical offset, showing spectral features associated with each component, (**F**) Cumulative and individual variance explained by each PCA mode. (**G**) Phase resolved spectra from PSD, (**H**) Traces of the phase angle spectra at wavenumbers 3500, 3020, and 1400 cm^{-1}, characterizing Components 1, 2, and 3, respectively, and (**I**) latent dimensions 1-3 from AE.

To further dissect the role of kinetic diversity in spectral decomposability, we expanded our benchmark dataset to include uncoupled systems with increasingly disparate time constants (from [1,1,1] to [1,5,25] s) and amplitude configurations (uniform, and two levels of skewed amplitudes: [1,5,10] and [1,10,100]). All systems were modulated using square wave input, which, as shown previously, maximizes τ observability by enforcing synchronized kinetic transients. Counterintuitively from knowledge in signal processing (23), increasing τ spread did not improve component separability. As summarized in Table S2, systems with nearly identical time constants yielded the lowest τ-ratio errors and the highest R_i-retrieval scores (PCA ε_τ = 11, R_i score = 1),

whereas systems with broader τ contrast (e.g., [1,5,25]) showed significant degradation (ε_τ = 259, R_i score = 0.69). These results defy classical signal processing expectations, where distinct time constants are presumed to simplify component resolution (23). This inversion stems from the structural constraints of linear decomposition methods like PCA. While increasing τ contrast may increase spectral variance, it also concentrates that variance into fewer principal axes, disproportionately amplifying slow-relaxing modes and suppressing faster dynamics. This biases PCA's orthogonal basis away from a faithful recovery of all kinetic components. ICA, which assumes statistical independence, suffers similarly due to its neglect of temporal causality and dynamic coupling. Consequently, both frameworks would fail to preserve dynamic balance when the system is skewed by broad τ or amplitude contrasts. Interestingly, the calculated AE models demonstrate more resilience to this skew. AE retrieval scores remain consistently high (0.95–0.99) under mild amplitude disparity and moderate τ spread but begin to deteriorate under strong skew (e.g., τ = [1,5,25], amps = [1,10,100]), indicating that even linear models can be challenged by severely asymmetric dynamics.

These results show that retrievability is maximized not when components are most different, but when their contributions are equitably distributed in both time and amplitude. When one component dominates the signal (due to high amplitude or long τ), decomposition algorithms skew toward it, suppressing weaker or faster dynamics, as expected. Hence, square input is not universally sufficient for perfect separability - it must be complemented by balanced system composition. This principle reflects a deeper distinction in decomposition philosophy. PCA and ICA maximize mathematical criteria (orthogonality, independence), not physical interpretability. Only in special cases, such as nearly equal τ and amplitude, do these align with the true structure of dynamic electrochemical systems. AE, being nonlinear and data-driven, offers more flexibility but still inherits limitations when signals are highly unbalanced. Ultimately, our findings challenge a central assumption of frequency decomposition: greater disparity does not guarantee better separability. In fact, homogeneous systems may be more retrievable, because they spread variance more evenly across dimensions, allowing decomposers to recover the full system rather than a biased projection.

Table S2. Overview of the calculated τ ratio error (ε_τ) (for PSD, and PCA), and the $R_{i\ retrieval\ score}$(for PCA, and AE) for 3 components, with varying τ ratios and equal amplitudes.

	τ ratio error (ε_τ)		$R_{i\ retrieval\ score}$	
τ values	PCA	AE	PCA	AE
		Amplitudes 1, 1, 1		
1, 1, 1	11	0.57	1.00	0.99
1, 2, 4	65	83587323	0.96	0.96
1, 3, 6	82	185	0.92	0.96
1, 5, 10	107	341	0.81	0.97
1, 5, 25	97	23	0.90	0.76
		Amplitudes 1, 5, 10		
1, 1, 1	15	166	1.00	0.99
1, 2, 4	9	5	0.95	0.98
1, 3, 6	43	905	0.91	0.97
1, 5, 10	61	6	0.84	0.95
1, 5, 25	259	337	0.69	0.79
		Amplitudes 1, 10, 100		
1, 1, 1	27	535	1.00	0.99
1, 2, 4	175597921	1313	0.93	0.99

1, 3, 6	67	182457080	0.87	0.89
1, 5, 10	51	9	0.80	0.87
1, 5, 25	22	99	0.85	0.91

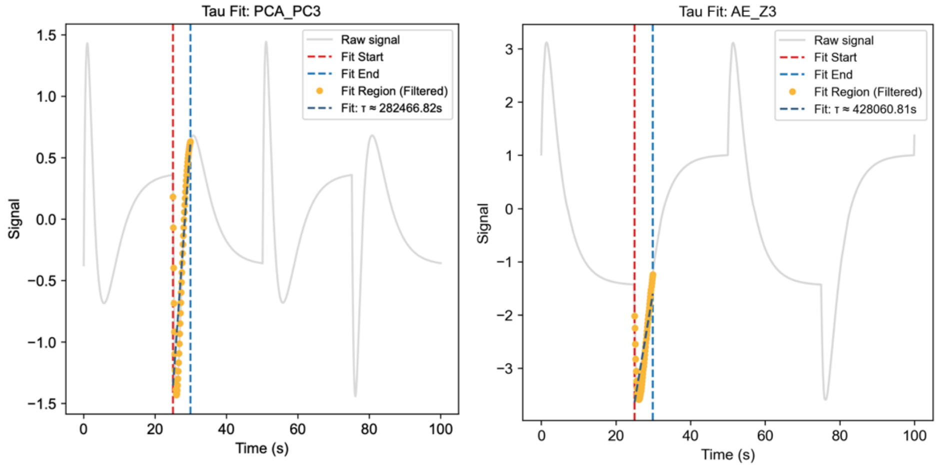

Figure S11. Fit τ_i values for the outliers from Table S2.

2.4.2 Nonlinear and Coupled systems

To extend the assessment of retrievability beyond linear and uncoupled systems and to reflect more realistic electrocatalytic interfaces where interactions between multiple dynamic components may render classical separability assumptions inadequate, we simulated a selection of complex systems according to the simulation framework described in Section S2A. Parameter sweeps included: (i) time constants (τ) spanning 1-10 s across components to test kinetic disparity; (ii) linear (α) and nonlinear (β) coupling strengths ranging from 0 to 0.5; (iii) nonlinear interaction types (thresholded, saturating, multiplicative, and stochastic); (iv) different numbers of active components (3–5); (v) spectral observability (fully, partially, or non-overlapping absorptive bands); (vi) waveform shape (square, sine, sawtooth, and constant), duration (1–100 s), and number of pulses (1, or fully extended); (vii) additive Gaussian noise levels (0–50%); and (viii) binary activation masks (off/off, off/on, on/on) per component to control responsiveness to modulation. These cases can be further expanded, and eventually be supplemented with, e.g., kinetic models specific to systems, but this goes beyond the scope of this work.

For each simulation, time-resolved response functions $R_i(t)$ were generated under structured electrochemical modulation, and their recoverability was evaluated via PCA and AE as described above.

Boxplot summaries of retrievability across four key system properties – input waveform, nonlinearity type, spectral overlap, and activation region – are shown in Figure S14. When evaluating retrievability across different nonlinear coupling types (Figure S14B), we found that saturating and threshold-type nonlinearities produced the most stable and accurate recovery of $R_i(t)$, while stochastic nonlinearities introduced moderate degradation. Interestingly, even under stochastic or sigmoidal distortion, PCA retained reasonably good retrieval scores, whereas AE performance was poorer, and more variable suggesting a higher sensitivity to noise and dynamic complexity due to its greater model flexibility. Spectral overlap among response components (Figures S14C) had less impact on retrievability than expected, though we note that the retrievability criteria could be further optimized to also fit for pure component retrievability in spectral space. Systems with fully overlapping spectra did not exhibit significantly worse performance than partially or non-overlapping cases. This indicates that temporal dynamics under modulation, rather than spectral orthogonality, primarily governs separability. The influence of activation masks (Figure S14D)

further revealed that the "off/on" regime supports improved separability. Systems in which only a subset of components responded to modulation achieved higher retrieval scores than fully activated ("on/on") systems, where dynamic entanglement was more pronounced. This effect was consistent in both PCA and AE, although AE exhibited greater variance in score distribution, likely reflecting its ability to internalize nonlinear mixtures but also its susceptibility to overfitting.

Figures S12 and S13 provide additional insight into the relationship between continuous simulation parameters, such as coupling strength (α, β), number of components, modulation duration, and noise, and retrieval performance. In PCA (Figure S12), no single parameter dominated retrievability, but moderate α and β values (~0.2–0.4) tended to support retrievability of $R_i(t)$, while high noise levels or large τ-ratios increased score variance as expected. AE (Figure S13) demonstrated stronger nonlinear trends; high retrievability was maintained for clean, low-dimensional conditions, but this deteriorated sharply in high-noise or highly coupled regimes, reflecting its data capacity constraints without explicit regularization.

In summary, both PCA and AE can extract meaningful latent structure from nonlinear, dynamically coupled systems, provided that input modulations are appropriately designed, and decompositions are interpreted with care. PCA, by virtue of its linearity and orthogonality constraints, offers robust, but sometimes overly rigid retrieval. AE can sometimes outperform PCA in selectively coupled or weakly nonlinear systems, but its increased flexibility introduces greater susceptibility to overfitting or misattribution in noisy, high-dimensional contexts. Importantly, no single waveform or decomposition method proved universally superior under all conditions (though PCA, on average, clearly performed better). We can conclude that retrievability arises from a confluence of factors, including modulation type, kinetic diversity, coupling strength, activation topology, and so forth, highlighting the need for deliberate, systems-informed design of experiments, but also analysis pipelines.

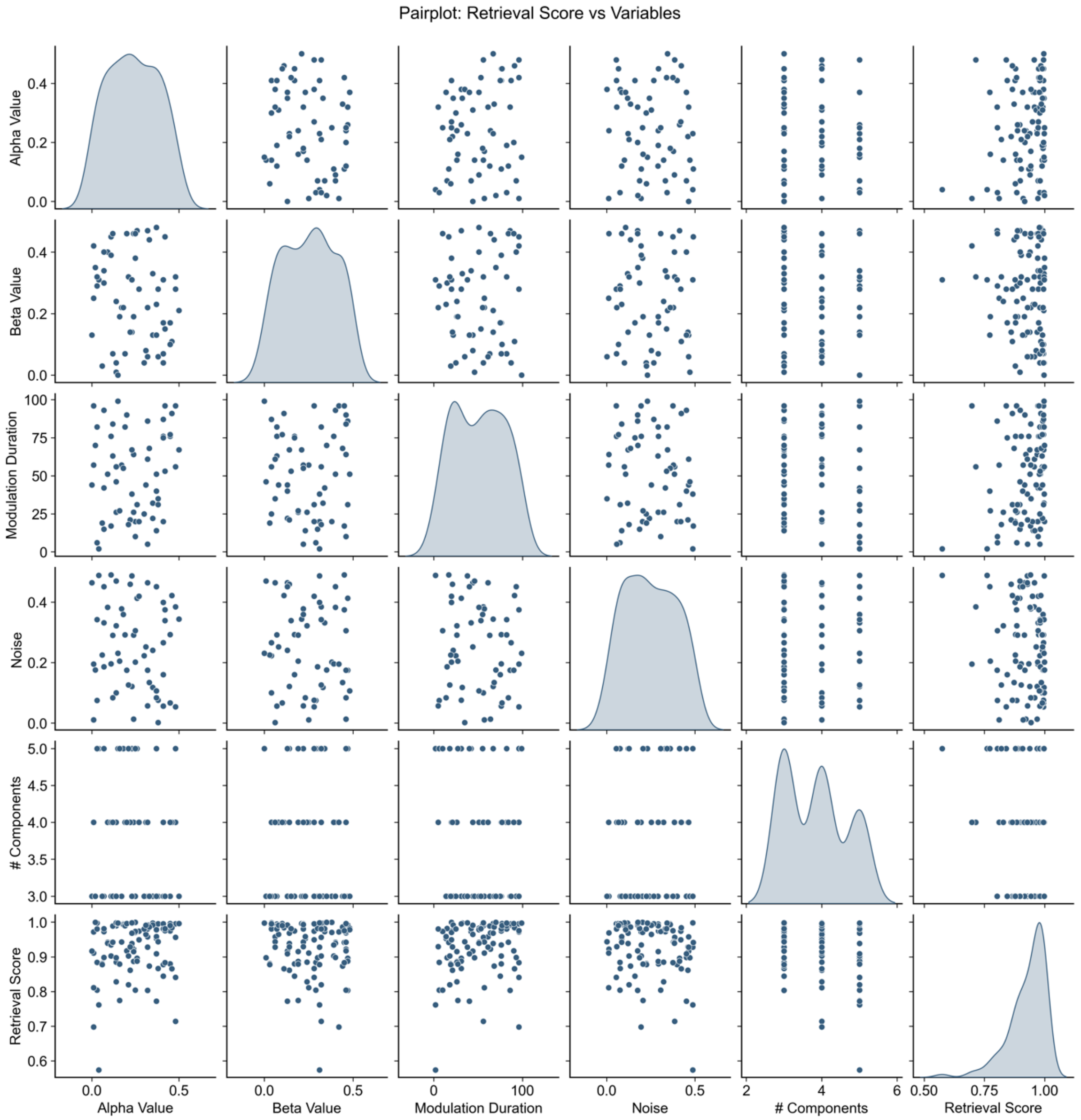

Figure S12. PCA Pairplot of Retrieval Score and Parameters. Scatterplot matrix showing the relationships between continuous simulation parameters (e.g., α, β, noise, modulation duration, number of components) and retrieval score.

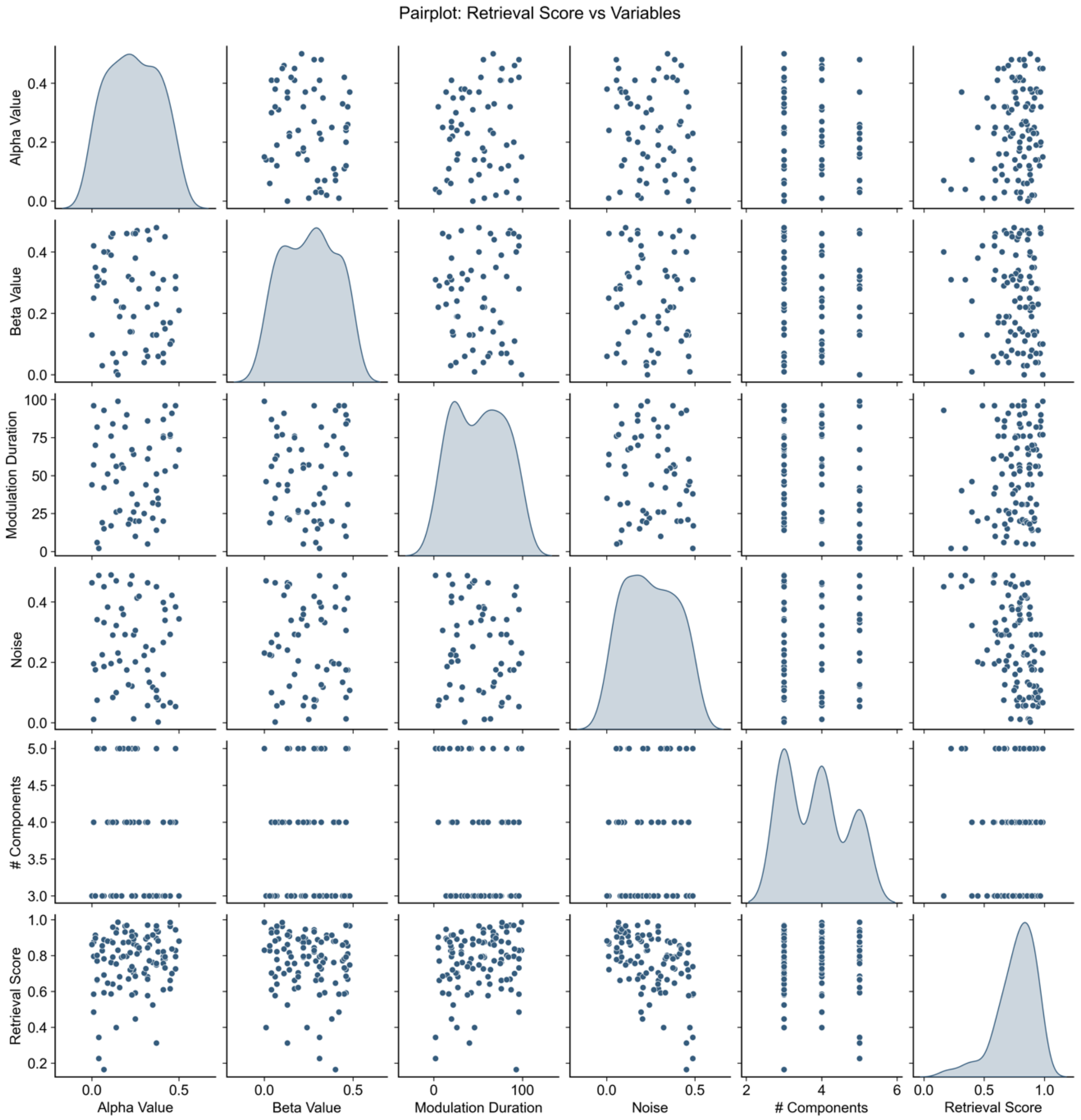

Figure S13. AE Pairplot of Retrieval Score and Parameters. Scatterplot matrix showing the relationships between continuous simulation parameters (e.g., α, β, noise, modulation duration, number of components) and retrieval score.

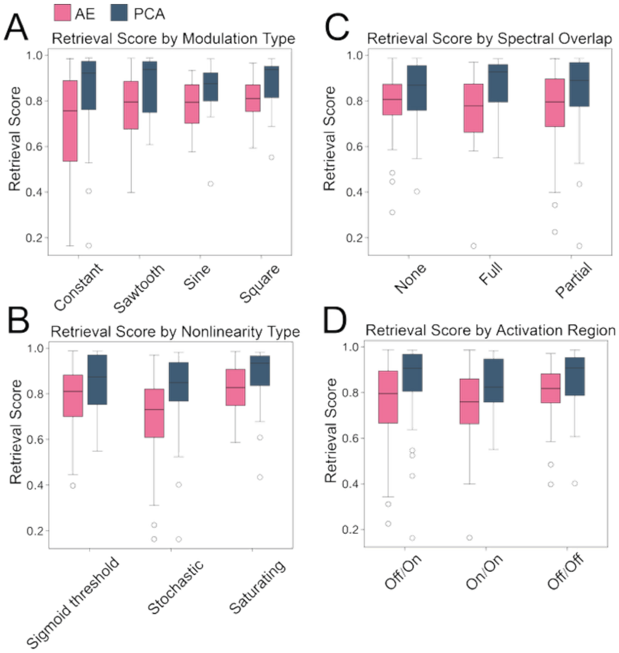

Figure S14. Expansion on the comprehensive benchmarking of PCA and AE approaches for resolving dynamic components in spectroelectrochemical systems, illustrating how coupling strength, modulation waveform, nonlinearity, and spectral structure influence the retrievability of kinetic information. (***A***) Boxplot comparing retrievability across different waveform inputs (sawtooth, sine, square, constant). While all modulations produce high median scores, sawtooth and square modulations offer slightly improved retrieval performance compared to sine or constant inputs, likely due to their smoother driving dynamics. (***B***) Comparison of response retrievability across systems with different nonlinear coupling functions. Saturating and thresholded nonlinearities support higher retrieval scores, while stochastic terms introduce variability but do not fully degrade the ability to resolve individual dynamics. (***C***) Retrieval performance as a function of spectral overlap among components. Partial and non-overlapping spectral cases yield comparable or better retrievability than fully overlapping cases, indicating that spectral orthogonality is not a strict requirement for disentanglement of dynamic responses under modulation. (***D***) Boxplot showing how the activation mask (off/off, off/on, on/on) affects the retrievability of underlying response functions $R_i(t)$. Fully activated systems (on/on) tend to show greater variability and lower median scores compared to selective or unmodulated regimes, due to increased nonlinear dynamic complexity, and off/on regimes show the greatest retrievability.

4. Supplementary Experimental Results

Below, experimental results derived from the performance of DRS experiments using 0.2 M $NaHCO_3$ under CO_2 saturation while alternating the potential between different potential windows (-0.05 to -0.4 VRHE, -0.4 to -0.8 VRHE, and -0.8 to -1.1 VRHE) are found. Experimental details on the performance of the experiment, including the spectroscopic set-up, can be found elsewhere (5).

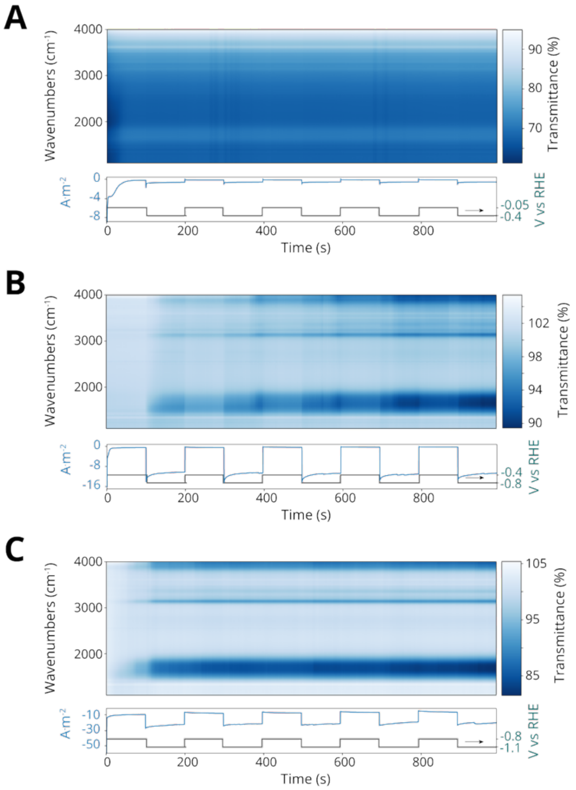

Figure S15. Time-resolved ATR-SEIRAS spectra (4000-1100 cm^{-1}) plotted as 2D maps during potential-pulsed CO_2RR experiments in 0.2 M $NaHCO_3$ electrolyte saturated with CO_2. Potential pulsing was applied between **(A)** -0.05 and -0.4 V_{RHE}, **(B)** -0.4 and -0.8 V_{RHE}, and **(C)** -0.8 and -1.1 V_{RHE}.

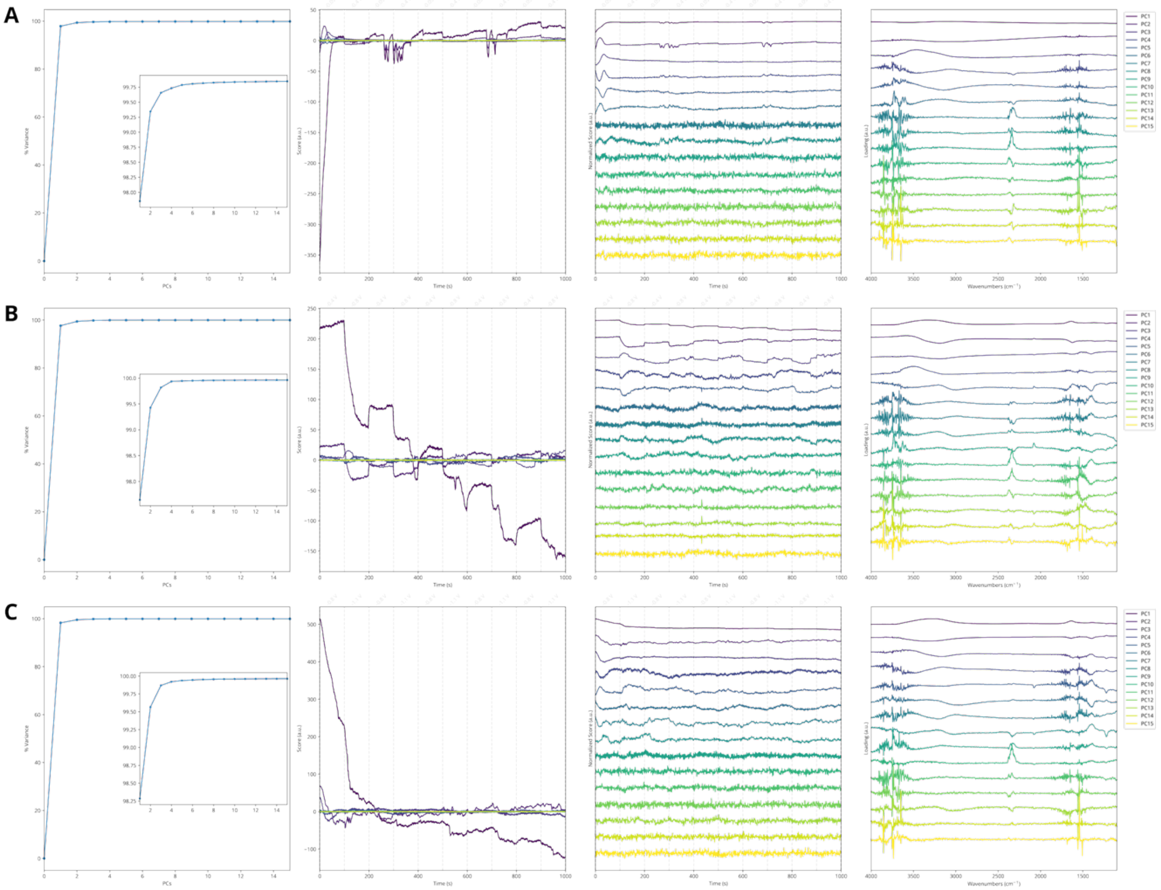

Figure S16. Cumulative variance explained (leftmost), evolution of the scores over time (left-center), evolution of the normalized scores over time (right-center) and eigenspectral (rightmost) resulted from the performance of PCA on the time-resolved ATR-SEIRAS spectra (4000-1100 cm^{-1}) obtained during potential-pulsed CO_2RR experiments in 0.2 M $NaHCO_3$ electrolyte saturated with CO_2. Potential pulsing was applied between **(A)** -0.05 and -0.4 V_{RHE}, **(B)** -0.4 and -0.8 V_{RHE}, and **(C)** -0.8 and -1.1 V_{RHE}.

4. Supplementary References

1. A. J. Bard, L. R. Faulkner, “Basic potential step methods” in *Electrochemical Methods: Fundamentals and Applications*, D. Harris, E. Swain, E. Aiello, Eds. (John Wiley & Sons, Hoboken, NJ, 2000), pp. 156–225.
2. A. J. Bard, L. R. Faulkner, “Basic Potential Step Methods” in *Electrochemical Methods: Fundamentals and Applications*, D. Harris, E. Swain, E. Aiello, Eds. (John Wiley & Sons, Hoboken, NJ, 2000), pp. 156–225.
3. G. R. Kepner, Saturation behavior: A general relationship described by a simple second-order differential equation *Theor. Biol. Med. Model.* **7** (2010).
4. A. Oppenheim, A. Willsky, S. Nawab, *Signals and Systems* (Prentice Hall, Hoboken, NJ, 1996).
5. D. Sinausia, *et al.*, Decoding double layer dynamics for CO_2 Electroreduction over Cu. *Angew. Chem. Int. Ed.* **64**, e202423177 (2025).
6. F. Lorenzutti, *et al.*, Microenvironment effects from first principles multiscale modeling of electrochemical CO_2 reduction. *Nat. Catal.* **8**, 905-918 (2025).
7. F. Geramipour, S. M. Mousavi Khoei, H. Shooshtari Gugtapeh, Effect of shaped waveform on structure and electrochemical corrosion behavior of pulse electrodeposited NiCu alloy coatings. *Surf. Coat. Technol.* **424**, 127643 (2021).
8. F. H. Pilz, P. Kielb, Cyclic voltammetry, square wave voltammetry or electrochemical impedance spectroscopy? Interrogating electrochemical approaches for the determination of electron transfer rates of immobilized redox proteins. *BBA Advances* **4**, 100095 (2023).
9. T. Yokoshima, *et al.*, Application of electrochemical impedance spectroscopy to ferri/ferrocyanide redox couple and lithium ion battery systems using a square wave as signal input. *Electrochim. Acta* **180**, 922–928 (2015).
10. V. De Coster, N. V. Srinath, P. Yazdani, H. Poelman, V. V. Galvita, Modulation engineering: Stimulation design for enhanced kinetic information from modulation-excitation experiments on catalytic systems. *ACS Catal.* **13**, 5084–5095 (2023).
11. K. J. Aoki, J. Chen, R. He, Potential step for double-layer capacitances obeying the power law. *ACS Omega* **5**, 7497–7502 (2020).
12. S. Wang, *et al.*, Electrochemical impedance spectroscopy. *Nat. Rev. Methods Primers* **1**, 41 (2021).
13. V. Vivier, M. E. Orazem, Impedance analysis of electrochemical systems. *Chem. Rev.* **122**, 11131–11168 (2022).
14. A. Ch. Lazanas, M. I. Prodromidis, Electrochemical impedance spectroscopy - a tutorial. *ACS Measurement Science Au* **3**, 162–193 (2023).
15. J. Krakowiak, W. Bącalski, G. Lentka, P. Peljo, P. Ślepski, Three modes of electrochemical impedance spectroscopy measurements performed on vanadium redox flow battery. *Sustain Mater Technol* **40**, e00957 (2024).
16. Y. Y. Liu, J. J. Slotine, A. L. Barabási, Observability of complex systems. *Proc. Natl. Acad. Sci. USA* **110**, 2460–2465 (2013).
17. O. Shamir, S. Sabato, N. Tishby, Learning and generalization with the information bottleneck. *Theor. Comput. Sci.* **411**, 2696–2711 (2010).
18. N. Tishby, F. C. Pereira, W. Bialek, The information bottleneck. arXiv [Preprint] (1999). https://arxiv.org/pdf/physics/0004057 (accessed 3 July 2025).
19. C. Hansen, *Rank-Deficient and Discrete Ill-Posed Problems: Numerical Aspects of Linear Inversion* (SIAM, 1998).
20. H. W. Knobloch, Observability of nonlinear systems, *Math. Bohem.* **131**, 411-418 (2006).
21. D. Gerbet, K. Röbenack, On global and local observability of nonlinear polynomial systems: A decidable criterion. *Automatisierungstechnik* **68**, 395–409 (2020).
22. R. Van Handel, Observability and nonlinear filtering. *Probab. Theory Relat. Fields.* **145**, 35–74 (2009).
23. A. V. Oppenheim, R. W. Schafer, J. R. Buck, *Discrete-time signal processing*, Marcia Horton, Ed., Second Edition (Prentice Hall, 1999).

24. A. Urakawa, T. Bürgi, A. Baiker, Sensitivity enhancement and dynamic behavior analysis by modulation excitation spectroscopy: Principle and application in heterogeneous catalysis. *Chem. Eng. Sci.* **63**, 4902–4909 (2008).
25. A. Urakawa, A. Baiker, Space-resolved profiling relevant in heterogeneous catalysis. *Top. Catal.* **52**, 1312–1322 (2009).
26. D. Baurecht, U. P. Fringeli, Quantitative modulated excitation Fourier transform infrared spectroscopy. *Rev. Sci. Instrum.* **72**, 3782–3792 (2001).
27. G. L. Chiarello, D. Ferri, Modulated excitation extended X-ray absorption fine structure spectroscopy. *Phys. Chem. Chem. Phys.* **17**, 10579–10591 (2015).
28. V. Marchionni, D. Ferri, O. Kröcher, A. Wokaun, Increasing the sensitivity to short-lived species in a modulated excitation experiment. *Anal. Chem.* **89**, 5801–5809 (2017).
29. F. Fleuret, "Machine learning" in The little book of deep learning, F. Fleuret (Université de Genève, 2023), pp. 11-19.
30. H. D. Nguyen, K. P. Tran, S. Thomassey, M. Hamad, Forecasting and anomaly detection approaches using LSTM and LSTM autoencoder techniques with the applications in supply chain management. *Int. J. Inf. Manage.* **57,** 102282 (2021).
31. G. E. Karniadakis, *et al.*, Physics-informed machine learning. *Nature Reviews Physics* **3**, 423-440 (2021).
32. M. Grossutti, *et al.*, Deep learning and infrared spectroscopy: Representation learning with a β-variational autoencoder. *J. Phys. Chem. Lett.* **13**, 5787–5793 (2022).
33. X.-Y. Guo, S.-E. Fang, A physics-informed auto-encoder based cable force identification framework for long-span bridges. *Structures* **60**, 105906 (2024).
34. Y. Wei, *et al.*, LSTM-autoencoder-based anomaly detection for indoor air quality time-series data. *IEEE Sens. J.* **23**, 3787–3800 (2023).
35. D. Sinausia, C. Vogt, Vogt Lab. GitHub. https://github.com/VogtLab. Deposited 2 July 2025.
